# Piezo1 restrains proinflammatory response but is essential in T-cell–mediated immunopathology

**DOI:** 10.1093/jleuko/qiae242

**Published:** 2024-11-04

**Authors:** Sung Hee Choi, Alicia Santin, Jay T Myers, Byung-Gyu Kim, Saada Eid, Suzanne L Tomchuck, Daniel T Kingsley, Alex Y Huang

**Affiliations:** Case Comprehensive Cancer Center, Case Western Reserve University School of Medicine, Cleveland, OH 44106, United States; Department of Pediatrics, Case Western Reserve University School of Medicine, Cleveland, OH 44106, United States; Department of Pathology, Case Western Reserve University School of Medicine, Cleveland, OH 44106, United States; Department of Pediatrics, Case Western Reserve University School of Medicine, Cleveland, OH 44106, United States; Case Comprehensive Cancer Center, Case Western Reserve University School of Medicine, Cleveland, OH 44106, United States; Department of Pediatrics, Case Western Reserve University School of Medicine, Cleveland, OH 44106, United States; Department of Pediatrics, Case Western Reserve University School of Medicine, Cleveland, OH 44106, United States; Department of Pediatrics, Case Western Reserve University School of Medicine, Cleveland, OH 44106, United States; Department of Pathology, Case Western Reserve University School of Medicine, Cleveland, OH 44106, United States; Case Comprehensive Cancer Center, Case Western Reserve University School of Medicine, Cleveland, OH 44106, United States; Department of Pediatrics, Case Western Reserve University School of Medicine, Cleveland, OH 44106, United States; Department of Pathology, Case Western Reserve University School of Medicine, Cleveland, OH 44106, United States; Center for Pediatric Immunotherapy, Angie Fowler Adolescent and Young Adult Cancer Institute, University Hospitals Rainbow Babies & Children’s Hospital, Cleveland, OH 44106, United States

**Keywords:** CD4^+^ T cell, colitis, EAE, functional polarization, gvHD, mechanosensation, piezo1, T-cell persistence

## Abstract

Piezo1 is a mechanosensitive, nonselective Ca^2+^ channel that is broadly expressed in CD4^+^ T cells. Using lineage-specific Piezo1 knockout mice (Piezo1^cKO^), we show that loss of Piezo1 in CD4^+^ T cells significantly increased IFNγ and IL-17 production in vitro under TH1 and TH17 polarizing conditions, respectively. Despite their intrinsic proinflammatory phenotype, Piezo1^cKO^ T cells are incapable of establishing disease in vivo in 3 separate adoptive transfer T-cell–mediated inflammatory mouse models, including experimental autoimmune encephalomyelitis, inflammatory bowel disease, and graft-vs-host disease. These phenomena coincided with a decreased effector memory (CD44^hi^CD62L^lo^) CD4^+^ T-cell pool derived from donor Piezo1^cKO^ T cells, an observation related to intrinsic T-cell fitness, as a cotransfer inflammatory bowel disease mouse model revealed a deficiency in the CD4^+^ effector memory population derived only from the naive Piezo1^cKO^ but a not coinfused Piezo1^WT^ CD4^+^ T-cell source. Taken together, our results support Piezo1 as restraining proinflammatory T-cell differentiation while contributing to the generation and persistence of the effector memory pool during CD4^+^ T-cell–mediated immunopathology.

## Teaser

Piezo1 restrains proinflammatory T-cell differentiation while contributing to the generation and persistence of the effector memory pool during CD4^+^ T-cell–mediated immunopathology.

## 1. Introduction

To carry out their specified functions, all cells must effectively perceive and respond to the biochemical and mechanical forces of their environment.^1,2^ This is accomplished, in part, through mechanosensing proteins that transduce physical information about the cell's surroundings to internal biochemical signaling cascades. This mechanosensation plays a key role in both the maintenance of normal cell physiology and the cell's ability to adapt to a changing microenvironment.^3,4^ Recent studies have demonstrated a central role for mechanosensation in 2 key cellular processes of T-cell biology: migration and activation.^5–10^ However, most models used to study T-cell biology neglect to address the importance of the mechanisms by which mechanical forces regulate T-cell function and differentiation.^11^

Calcium (Ca^2+^) influx is a crucial early step during T-cell activation necessary to direct the signaling cascades that ultimately control T-cell responses such as cytokine secretion, T-cell proliferation, and effector function differentiation of T helper (T_H_) cells.^12–15^ Dysregulated calcium responses in T cells have been associated with several autoimmune and inflammatory diseases, including systemic lupus erythematosus, rheumatoid arthritis, psoriasis, and inflammatory bowel disease (IBD).^16^ Furthermore, inhibition of Ca^2+^ influx, through the deletion of Ca^2+^ release-activated Ca^2+^ (CRAC) channels or Ca^2+^ sensors, ameliorated pathogenic T_H_17 cell-mediated intestinal inflammation.^17,18^ While CRAC channels have been described as the major source for Ca^2+^ entry into T cells, several additional families of channels have been implicated in regulating T-cell biology.^13,19^ Piezo1 is a mechanosensitive cation channel that has vital roles in both migration and cell fate in a variety of cell types.^20–22^ Piezo proteins are uncommonly large cation channels that exist as a 3-bladed, propeller-shaped homotrimeric structure with a central channel and cap located on the C terminal of each monomer.^23–27^ Due to its shape, Piezo channels locally deform lipid membranes into a concave dome-like shape in their closed conformation. Under lateral membrane tension, the Piezo structure flattens, lifting the “cap” and opening the channel to facilitate influx of extracellular Ca^2+^.^28–32^ Piezo1 is highly expressed in tissues with high mechanical pressures and is expressed across a wide range of immune cell subsets, including all populations of T cells.^33–36^ Although many aspects of T-cell biology require effective mechanosensing and Piezo1 is known to be highly expressed in T cells, little is known about the role(s) and mechanism(s) of Piezo1 in regulating T-cell migration, activation, differentiation, or cell fate decisions.

Utilizing a mouse model with CD4-Cre–driven T-cell–specific conditional deletion of Piezo1, we previously demonstrated that functional Piezo1 in CD4^+^ T cells, not CD8^+^ T cells, is indispensable for effective antitumor immunity.^34^ This and other studies point to a disproportionately important role of Piezo1 in CD4^+^ T-cell biology.^37^ In the current study, we further investigate the role of Piezo1 in CD4^+^ T-cell function both in vitro and in vivo. We observed that, in the absence of Piezo1 in CD4^+^ T cells, the production of proinflammatory cytokines is significantly increased during T-cell activation in vitro. Specifically, IFNγ- and IL-17–producing CD4^+^ T cells under T_H_1 and T_H_17 polarizing conditions, respectively, are significantly increased compared to their wild-type counterparts. Despite these in vitro findings, however, the absence of Piezo1 prevents autoimmune and alloimmune inflammation in in vivo models of experimental autoimmune encephalomyelitis (EAE), IBD, and graft-vs-host disease (GvHD) with a decrease in the CD4^+^ T-cell effector memory (T_EM_) population. Collectively, our data demonstrate that Piezo1 contributes to the pathogenesis of CD4^+^ T-cell–mediated autoimmune disease by restraining CD4^+^ T-cell activation and maintaining effector memory T cells.

## 2. Methods

### 2.1 Mice

CD4-Cre (Stock #22071), Piezo1^WT^ (Piezo1^fl/fl^, Stock #29213), Rag1^−/−^ (Stock #2216), C57BL/6J (Stock #664), C57BL/6J CD45.1 (Stock #2014), and BALB/cJ (Stock #651) mice were obtained from the Jackson Laboratory. To generate Piezo1^cKO^ mice lacking Piezo1 specifically in T cells, Piezo1^WT^ were crossed with CD4-Cre. Mice were housed in a pathogen-free facility. All animal experiments were previously approved by the Institutional Animal Care and Use Committee at Case Western Reserve University (Protocol #: 2016-0067).

### 2.2 Intracellular Ca^2+^ measurements

Intracellular Ca^2+^ measurements were performed as previously reported.^34^ Bulk T cells were isolated from the spleens of Piezo1^WT^ and Piezo1^cKO^ mice using the negative selection of B-220 (Invitrogen) and stimulated overnight with anti-CD3/anti-CD28 coated plates. The next day, cells were labeled with anti–CD4-APC/Cy7 (BioLegend, RM4-5), anti–CD8-APC (BioLegend, 53-6.7), and 5 µM Fluo4-AM for 15 min at 37 °C. The cells were then resuspended in stimulation buffer containing 5 µm ionomycin, 25 µM Yoda1, or DMSO in the presence or absence of either 2 mM CaCl2 or EGTA and analyzed by flow cytometry.

### 2.3 In vitro T-cell proliferation assay

Mouse CD4^+^ T cells were isolated from spleens using the CD4^+^ T-cell isolation kit (Miltenyi Biotec), labeled with carboxyfluorescein succinimidyl ester (CFSE) (1 µM; Invitrogen), and then plated in a 24-well with anti-CD3/anti–CD8-coated beads (Dynabeads Mouse T-Activator CD3/CD28; Gibco). Then, 72 h later, CD4^+^ T-cell division was determined by flow cytometry.

### 2.4 In vitro T-cell activation and differentiation

CD4^+^ T cells were purified from the spleens of Piezo1^WT^ and Piezo1^cKO^ mice and stimulated with anti-CD3/anti–CD8-coated beads (Dynabeads Mouse T-Activator CD3/CD28; Gibco). For differentiation of CD4^+^ T cells into T_H_1, T_H_17, and Treg cells, T cells were stimulated for 3 d with anti-CD3 (1 μg/mL)/anti-CD8 (0.5 μg/mL)–coated plates and IL-12 (10 ng/mL), anti–IL-4 antibody (1 μg/mL), IL-2 (5 ng/mL), and IFNγ (20 ng/mL) for T_H_1; IL-6 (20 ng/mL), TGF-β (1 ng/mL), anti–IL-4 antibody (1 μg/mL), anti-IFNγ antibody (1 μg/mL), and anti–IL-2 antibody (1 μg/mL) for T_H_17; and anti-IFNγ antibody (1 μg/mL), anti–IL-4 antibody (1 μg/mL), and TGF-β (2 ng/mL) for Treg. For cytokine expression, cells were stimulated with the BioLegend T-cell activation cocktail containing ionomycin, phorbol myristate acetate (PMA), and brefeldin A for 5 h and analyzed by flow cytometry or quantitative polymerase chain reaction (qPCR) analysis.

### 2.5 RNA sequencing

Constructed sequencing libraries were subject to sequencing with an Illumina Novaseq 6000 platform (paired-end, 150 bp). RNA sequencing was performed and analyzed by LC Science as previously published.^38^ Before assembly, reads containing sequencing adaptors, reads containing sequencing primers, and sequences with a q quality score lower than 20 were removed. The cleaned sequencing reads were aligned to the reference genome using the HISAT2 package. Multiple alignments were allowed for each read sequence (up to 20 by default), with a maximum of 2 mismatches allowed. HISAT2 also built a database of potential splice junctions. Aligned reads of individual samples were assembled using StringTie. Transcriptomes from all samples were then merged to reconstruct a comprehensive transcriptome using a proprietary in-house Perl script of LC Sciences, Cutadapt. Following transcriptome reconstruction, Fragments Per Kilobase of transcript per Million mapped reads (FPKM) reads were evaluated by StringTie. Normalization of the raw read counts and the differential expression analyses were performed using the DESeq2 package. Genes were considered differentially expressed if they had an adjusted *P* value (using Benjamini–Hochberg corrections) <0.05 and log_2_ fold change >0.3 or <0.3. Shrunken log_2_ fold changes were computed using the ashr estimator from the ashr package. Genes were annotated using the org.Hs.eg.db database package. Gene set enrichment analysis (GSEA) was performed using the Broad Institute tool GSEA v.2.2.1. Gene sets were tested against included the H and C7 gene sets from the Molecular Signatures Database (MSigDB). RNA sequencing (RNA-seq) data used in this study have been deposited in the SRA database (NCBI) under accession code PRJNA1081599.

### 2.6 Dextran sulfate sodium (DSS)-induced colitis

Experimental colitis was induced by administration of 2% (w/v) DSS (MP Biomedicals) in drinking water for 7 d, followed by a regimen of 3 d of normal water. The disease activity index (DAI) score was calculated by combining the score of stool consistency (scale of 0 to 3), weight loss (scale of 0 to 3), and gross bleeding (scale of 0 to 3). The survival rate was measured for 10 d. For pathology, colon tissue samples were washed with phosphate-buffered saline (PBS), cut longitudinally, and then formalin fixed and paraffin embedded. Lamina propria for fluorescence-activated cell sorting (FACS) and qPCR and epithelial cells for Western blot were obtained using the lamina propria dissociation kit (Miltenyi Biotec).

### 2.7 T-cell adoptive transfer-induced colitis

Spleen cells from Piezo1^WT^ or Piezo1^WT CD4cre^ mice were stained with anti–CD4-APC (clone RM4-5, BioLegend) and anti–CD45RB-PE (C363-16A, BioLegend) and sorted on a FACSAria (Becton Dickinson). The sorted naive CD4^+^CD45RB^hi^ cells were >99% pure. Five matched Rag1^−/−^ recipient mice received 5 × 10^5^ CD4^+^CD45RB^hi^ T cells by intraperitoneal (i.p.) injection. Rag1 mice were assessed once a week for weight loss and other symptoms of distress over a course of 13 wk, and intestinal tissues were collected for histologic analysis and measurements of cytokines. For CD4^+^ T-cell cotransfer experiments, Rag1^−/−^ mice were adoptively cotransferred with naive CD4^+^ T cells isolated from Piezo1^WT^ mice (CD45.1) and Piezo1^cKO^ mice (CD45.2) at a ratio of 1:1 (5 × 10^5^:5 × 10^5^). Recipients were sacrificed 2, 4, or 13 wk after transfer. The number and subpopulations of C57BL/6 CD4^+^ T cells (CD45.1^+^) and Piezo1^cKO^ CD4^+^ T cells (CD45.2^+^) in the spleen (SPL), mesenteric lymph node (mLN), and colon [lamina propria (LP)] at 2, 4, and 13 weeks postinjection were analyzed via flow cytometry. For intracellular staining of cytokines, lymphocytes from spleen and lamina propria were activated with plate-bound anti-CD3 and anti-CD28 antibodies for 48 h and restimulated with PMA and ionomycin for the last 5 h in the presence of Golgi stop solution before flow cytometry staining.

### 2.8 Graft-vs-host disease

BALB/cJ host mice (H-2d) were conditioned by myeloablative total body irradiation with a split 2 doses of 8.0 Gy total using a Gammacell 40 Exactor System (Best Theratonics). At 3 h after total body irradiation, mice were injected retro-orbitally with 5 × 10^6^ Piezo1^WT^ or Piezo1^cKO^ BM cells (H-2b) with 1 × 10^6^ Piezo1^WT^ or Piezo1^cKO^ total T cells (H-2b). Animals were treated with antibiotic drinking water (sulfamethoxazole and trimethoprim; Hi-Tech Pharmacal) for 7 d before and after allogeneic hematopoietic cell transplantation (allo-HCT) to prevent unspecific infections. Transplanted mice were assessed daily for 5 clinical parameters (weight loss, posture, fur, skin, and activity). The score of each parameter is 0 to 2 (total score 0 to 10). Survival was monitored daily. Mice were sacrificed when GvHD symptoms reached our end-point scoring criteria.

### 2.9 Experimental autoimmune encephalomyelitis

For active EAE, Piezo1^WT^ or Piezo1^cKO^ mice were immunized by a subcutaneous injection of MOG_35–55_ in Complete Freud's Adjuvant (CFA) at 2 sites totaling 0.2 mL/mouse (Hooke Labs). Then, 200 ng pertussis toxin (Hooke Labs) was injected (i.p.) 2 and 24 h after immunization. Scoring and assessment of mice began on day 7. To promote adoptive T-cell transfer-induced EAE (AT-EAE), Piezo1^WT^ or Piezo1^cKO^ mice were immunized with MOG_35–55_ peptide. Eleven days later, bulk splenocytes were activated in vitro with 20 μg MOG_35–55_ peptide and rmIL-23 (20 ng/mL) for 3 d. A total of 20 × 10^6^ donor cells were injected (i.p.) into each wild-type C57BL/6 (CD45.1) recipient mouse. Scoring and assessment of recipient mice began on day 5. The clinical symptoms of EAE were assessed for each animal daily according to the following criteria: no clinical signs (0), partially limp tail (0.5), paralyzed tail (1), loss of coordinated movement and hindlimb paresis (2), 1 hindlimb paralyzed (2.5), both hindlimbs paralyzed (3.0), hindlimbs paralyzed and weakness in forelimbs (3.5), forelimbs paralyzed (quadriplegia) (4), and moribund (5). Mice would be euthanized if a score was greater than 4.0 for 48 h, as per Institutional Animal Care and Use Committee policy.

### 2.10 OVA_323–339_ immunization

OT-II transgenic Piezo1^WT^ or Piezo1^cKO^ mice were immunized by intradermal injection of OVA_323–339_ in CFA at 1 site in the base of the tail, totaling 0.05 mL/mouse (Hooke Labs). Spleens and draining lymph nodes (inguinal) were harvested at 10 and 44 days postinjection. Flow cytometry immediately stained and analyzed a portion of the cells for activation, exhaustion, and memory markers. The rest of the cells were plated with irradiated (3,000 Rads) splenocytes from a C57Bl/6 mouse that were pulsed with OVA_323–339_ (5 ng/μL). Then, 72 h later, cells were stained and analyzed by flow cytometry as above.

### 2.11 Flow cytometry

Antibodies (Abs) against intracellular and extracellular markers were purchased from eBioscience, BD Pharmingen, or BioLegend and are as follows: anti-mouse CD3e (clone 145.2C11), anti-mouse CD4 (clone RM4-5), anti-mouse CD8α (clone 53.6.7), anti-mouse CD25 (clone PC61), anti-mouse/human CD44 (clone IM7), anti-mouse CD45.2 (clone A20), anti-mouse CD62L (clone MEL-14), anti-mouse CD69 (clone H1.2F3), anti-mouse CD95 (clone SA367H8), anti-mouse CCR5 (clone HM-CCR5), anti-mouse CCR7 (clone 4B12), anti-mouse CTLA-4 (clone UC10-4B9), anti-mouse FOXP3 (clone FJK-16 s), anti-mouse IFNγ (clone XMG1.2), anti-mouse IL-17a (clone TC11-18H10.1), anti-mouse LAG-3 (clone C9B7W), anti-mouse PD-1 (clone 29F.1A12), anti-mouse TIGIT (clone A17200C), and anti-mouse TNFα (clone MP6-XT22). All antibodies were used at 1:100 dilution. Additionally, live/dead discrimination was performed using 7-AAD (BioLegend) or the Zombie NIR viability kit (BioLegend).

Cells were stimulated with T-cell activation cocktail containing Brefeldin A (BioLegend) for 5 h, washed in ice-cold FACS buffer [PBS/2.5 mM EDTA/0.1% bovine serum albumin (BSA)], and incubated in blocking buffer (1:400 anti-mouse CD16/32 in FACS buffer) for 15 min on ice. Cells were washed and Abs were added for 30 min on ice. The stained samples were then washed twice with ice-cold FACS buffer and run on a BD Accuri C6 or CytoFlex flow cytometer (Beckman Coulter). The data were then analyzed using the BD Accuri C6, FlowJo, or CytoExpert software (Beckman Coulter). For intracellular cytokine staining, cells were incubated with Fixation/Permeabilization (eBioscience) for 1 h at room temperature and washed with FACS buffer. The cells were incubated with blocking buffer (2% normal mouse serum in FACS buffer) for 15 min at room temperature, and intracellular Abs were added for 30 min at room temperature before washing and running on the flow cytometer. Flow gating strategies are provided (Supplementary Figs. 2, 4, and 7).

### 2.12 Immunohistochemistry

For hematoxylin and eosin (H&E) staining, spinal cords or colon were fixed in 10% formalin, paraffin embedded, and stained with H&E using standard protocols. For immunohistochemistry, slides were incubated with primary antibodies CD3 (MA5-14524, Invitrogen). Antibodies were detected using Rabbit-on-Rodent HRP polymer (RMR622G, BioCare) and visualized using the Betazoid DAB chromogen kit (SK-4103-100, Vector Laboratories). Before examination, the nuclei were counterstained with Hematoxylin (H-3404-100, Vector).

### 2.13 Western blotting

For Western blotting, colon mucosa was obtained from scrapings of full-length colon and lysed in lysis buffer (150 mM NaCl; 20 mM Tris-Cl, pH 7.5; 1 mM PMSF; 1 mM Na_3_VO_4_; 25 mM NaF; 1% aprotinin; 10 μg/mL leupeptin). Proteins were fractionated by sodium dodecyl sulfate–polyacrylamide gel electrophoresis and transferred onto a nitrocellulose membrane (Invitrogen). The membranes were blocked with 5% BSA for 1 h and then incubated in buffer containing the primary antibody overnight at 4 °C. Membranes were incubated with horseradish peroxidase–conjugated secondary antibodies for 1 h. Western blots were visualized by enhanced chemiluminescence (ECL).

### 2.14 qPCR

Total RNA was isolated using Trizol reagent (Invitrogen). For qPCR, we used 1 μg RNA for complementary DNA (cDNA) using a High-Capacity cDNA synthesis kit (Applied Biosystems). A BioRad CFX96 Real-Time System C1000 Thermal Cycler was used to analyze the amplified cDNA. Primers for *GAPDH* were used as the housekeeping gene to normalize gene expression in wild-type tissue. The relative messenger RNA (mRNA) expression was calculated with comparative cycle threshold analysis (ΔΔCT).

### 2.15 Statistical analysis

Two-tailed, unpaired Student’s *t*-test was used to compare differences in Piezo1^WT^ or Piezo1^cKO^ mice. Two-way analysis of variance was applied to evaluate body weight changes and the DAI. Bonferroni's post hoc test was performed when applicable. All data are presented as the mean ± SEM. Statistical significance was accepted as a *P* value less than or equal to 0.05, with *P < 0.05, **P < 0.01, ***P < 0.001, and ****P < 0.0001. All statistical analyses were performed using GraphPad Prism (GraphPad Software).

## 3. Results

### 3.1 Piezo1 restrains CD4^+^ T-cell polarization in vitro

**Fig. 1.**
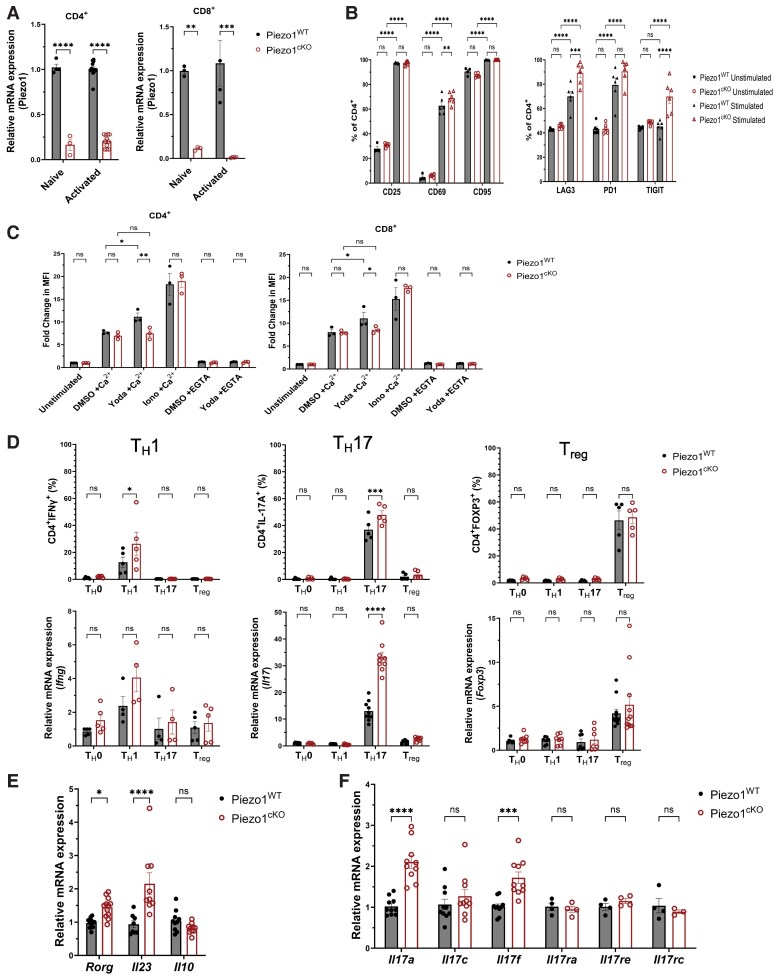
Piezo1 controls the T-cell polarization in vitro. (A) *Piezo1* mRNA transcript expression in wild-type mice (Piezo1^WT^) and CD4-Cre–mediated Piezo1 conditional knockout mice (Piezo1^cKO^). CD4^+^ T and CD8^+^ T cells were isolated from spleen of Piezo1^WT^ and Piezo1^cKO^ mice, and *Piezo1* mRNA in naive and anti-CD3/anti-CD28 antibody activated CD4^+^ and CD8^+^ T cells was measured by qPCR. (Naive CD4^+^ T cell, *n* = 4; activated CD4^+^ T cell, *n* = 12; naive CD8^+^ T cell, *n* = 3; activated CD8^+^ T cell, *n* = 4). (B) Frequency of activation and exhaustion markers of activated CD4^+^ T cells as in C. (C) Quantification of the calcium flux of CD4^+^ T cells from Piezo1^WT^ and Piezo1^cKO^ mice. T cells were isolated from spleens of Piezo1^WT^ and Piezo1^cKO^ mice, stimulated with anti-CD3/anti-CD28 antibodies, and stained with Fluo4-AM. Cells were then treated with ionomycin or Yoda1 in the presence or absence of CaCl_2_ or EGTA, and the resulting calcium flux was measured by flow cytometry (*n* = 3). (D) Quantification of T cells isolated from Piezo1^WT^ or Piezo1^cKO^ mice was activated under T_H_1, T_H_17, and Treg polarizing conditions for 3 d. Cells were then restimulated with PMA/ionomycin for 5 h; stained for intracellular contents of IFNγ, IL-17, and FOXP3; and analyzed by flow cytometry (*n* = 5 from 2 separate experiments). Transcripts were normalized to the expression of *GAPDH*, and mRNA from undifferentiated naive T cells (T_H_0) served as the baseline for gene expression (*Ifnγ*, *n* = 4 to 5; *Il-17*, *n* = 8 to 10; *Foxp3*, *n* = 7 to 11). (E) mRNA expression of IL-17 isoforms under T_H_17 polarizing condition. *Rorγ*, *Il-23*, and *Il-10* expression in T cells following 3 d under T_H_17 polarizing conditions were measured by qPCR. Transcripts were normalized to the expression of *GAPDH*, and mRNA from T cells of wild-type (Piezo1^WT^) served as the baseline for gene expression (*n* = 8 to 12). (F) Transcript expression of T_H_17 family subsets under T_H_17 polarizing conditions. CD4^+^ T cells were isolated from the spleen of Piezo1^WT^ or Piezo1^cKO^ mice and activated with anti-CD3/anti-CD28 antibodies for 3 d under T_H_17 polarizing conditions. Transcripts were normalized to *GAPDH*, and mRNA from Piezo1^WT^ T cells served as the baseline for gene expression (*n* = 3 to 10). Data were analyzed by 2-way analysis of variance with Šídák post hoc test (A, C, E–G), Fisher least significant difference post hoc test (B), or Tukey post hoc test (D). All bar graphs are shown as the mean ± SEM with *P < 0.05, **P < 0.01, ***P < 0.001, and ****P < 0.0001.

Piezo1 is a recently identified mechanosensitive nonselective Ca^2+^-permeable cation channel that is broadly expressed in mammalian cells.^23,26–28,32,33^ We have generated a T-cell–specific Piezo1 knockout mouse model (Piezo1^cKO^) by crossing Piezo1-floxed mice (Piezo1^WT^) to CD4-Cre mice. CD4^+^ T cells were isolated from the spleens of Piezo1^cKO^ and Piezo1^WT^ control mice. mRNA expression of Piezo1 in both naive and activated CD4^+^ T cells was evaluated by qPCR, which confirmed that Piezo1 mRNA transcript was significantly diminished in both CD4^+^ T cells and CD8^+^ T cells of Piezo1^cKO^ (Fig. 1A). To ensure functional disruption of Piezo1 in CD4^+^ T cells, we evaluated changes in Ca^2+^ influx using the synthetic small-molecule Piezo1-specific agonist, Yoda1.^39^ Yoda1 significantly increased the intracellular Ca^2+^ concentration in Piezo1^WT^ CD4^+^ T cells but failed to induce Ca^2+^ responses in Piezo1^cKO^ CD4^+^ T cells. In contrast, no differences were seen in the presence of ionomycin, confirming the specific functional loss of Piezo1 in Piezo1^cKO^ T cells (Fig. 1C). Similarly, Yoda1 was unable to increase intracellular Ca^2+^ concentration in Piezo1^cKO^ CD8^+^ T cells (Fig. 1C). Next, we examined the effects of Piezo1 deletion on T-cell proliferation, activation, and differentiation. Piezo1^cKO^ CD4^+^ T cells did not exhibit a deficiency in proliferation or activation as measured by CFSE and expression of CD25, CD95, and PD1 (Supplementary Fig. 1A and B). However, a significantly higher frequency of CD69, LAG3, and TIGIT was observed following 3 d of in vitro stimulation of Piezo1^cKO^ CD4^+^ T cells (Fig. 1B). Interestingly, when naive CD4^+^ T cells were stimulated under T_H_1, T_H_17, and Treg differentiation conditions in vitro, loss of Piezo1 significantly increased the frequency of IFNγ- and IL-17A–producing CD4^+^ T cells in T_H_1 and T_H_17 polarizing conditions, respectively, while the frequency of FOXP3-expressing Treg cells remained the same between T cells isolated from Piezo1^cKO^ and Piezo1^WT^ (Fig. 1D). These results suggest that Piezo1 plays a role in restraining T_H_1 and T_H_17 polarization as well as T-cell overactivation and exhaustion. To better understand how Piezo1 regulates T_H_17 T-cell differentiation, we analyzed factors that contribute to T_H_17 differentiation. Levels of *Ror*γ and *Il-23* but not *Il-10* were significantly increased in CD4^+^ T cells from Piezo1^cKO^ (Fig. 1E).^40^ In the IL-17 subfamily, *Il-17a* and *Il-17f* mRNA were significantly elevated in Piezo1^cKO^ CD4^+^ T cells under T_H_17 polarizing conditions (Fig. 1F).

### 3.2 Piezo1 disruption enhances a proinflammatory signature in T cells

**Fig. 2.**
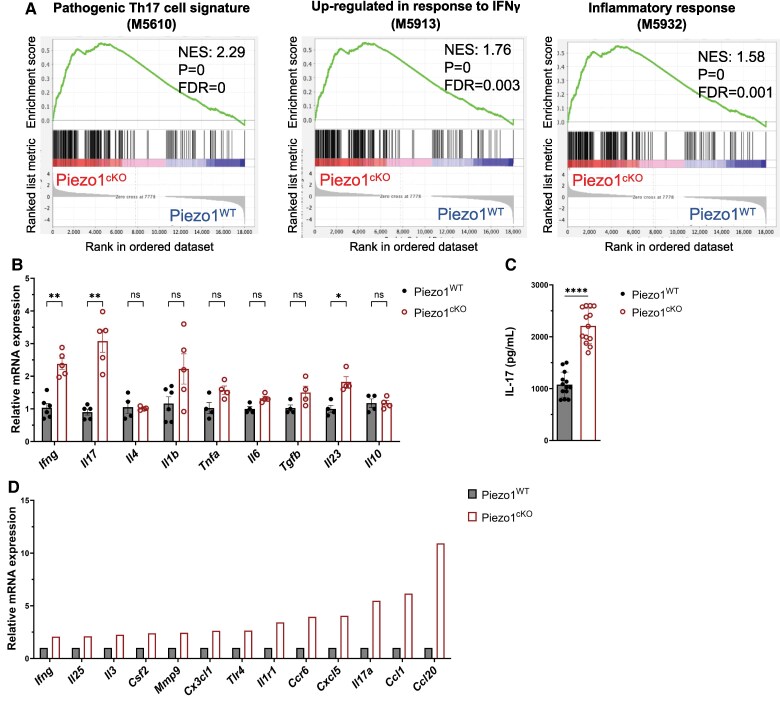
Deletion of Piezo1 enhances a proinflammatory signature in T cells. Purified naive CD4^+^ T cells of Piezo1^cKO^ and Piezo1^WT^ were activated with anti-CD3/anti-CD28 antibodies for 3 d and (A) RNA sequencing analysis was performed. Hallmark pathways from GSEA revealed a significantly upregulated pathogenic T_H_17 cell signature, genes upregulated in response to IFNγ, and inflammatory response gene sets in Piezo1^cKO^ CD4^+^ T cells compared with Piezo1^WT^ (*n* = 3). (B) Piezo1^cKO^ CD4^+^ T cells have an increased transcript expression of proinflammatory cytokines compared to Piezo1^WT^ as determined by qPCR. Transcripts were normalized to *GAPDH* and mRNA from Piezo1^WT^ T cells (*n* = 4 to 6). (C) IL-17 production in activated CD4^+^ T cells. CD4^+^ T cells were isolated from the spleen of Piezo1^WT^ or Piezo1^cKO^ mice and activated with anti-CD3/anti-CD28 antibodies for 3 d in vitro. IL-17 in the media was measured by an enzyme-linked immunosorbent assay (*n* = 13). (D) Increased T_H_17 response in Piezo1^cKO^ CD4^+^ T cells compared to Piezo1^WT^ CD4^+^ T cells was determined by the T_H_17 Response RT2 Profiler PCR array. Data were analyzed by 2-way analysis of variance with Šídák post hoc test (B, C). All bar graphs are shown as the mean ± SEM with *P < 0.05, **P < 0.01, ***P < 0.001, and ****P < 0.0001.

To further characterize the proinflammatory profile of Piezo1^cKO^ CD4^+^ T cells, we performed an RNA-seq analysis of CD4^+^ T cells after 3 d of stimulation with plate-bound anti-CD3 and anti-CD28 antibodies. GSEA indicated that the expression of genes related to pathogenic T_H_17 cells, IFNγ response, and inflammatory responses were all significantly increased in Piezo1^cKO^ CD4^+^ T cells compared to Piezo1^WT^ CD4^+^ T cells (Fig. 2A). We confirmed the increased transcription of proinflammatory cytokines, including *Ifnγ*, *Il-17*, *Tnfα*, *Il-1β*, and *Il-23* in activated Piezo1^cKO^ CD4^+^ T cells compared with Piezo1^WT^ counterparts by qPCR (Fig. 2B). Additionally, activated Piezo1^cKO^ CD4^+^ T cells secreted a significantly increased amount of IL-17 after 3 d of stimulation in vitro (Fig. 2C). Next, using a RT² Profiler PCR T_H_17 response array, we further confirmed that Piezo1^cKO^ CD4^+^ T cells have increased T_H_17 transcript responses compared with that of Piezo1^WT^; 13 genes were increased more than 2-fold, with *Ccl20*, *Ccl1*, and *Il-17a* being elevated 10.9-, 6.1-, and 5.5-fold, respectively, in Piezo1^cKO^ CD4^+^ T cells compared to Piezo1^WT^ control (Fig. 2D). Taken together, these data suggest that Piezo1 expression in CD4^+^ T cells could play a key role in the pathogenesis of CD4^+^ T-cell–mediated inflammatory diseases via restraining pathogenic T_H_1 and T_H_17 cell development.

### 3.3 Deletion of Piezo1 in T cells accelerates DSS-induced colitis

**Fig. 3.**
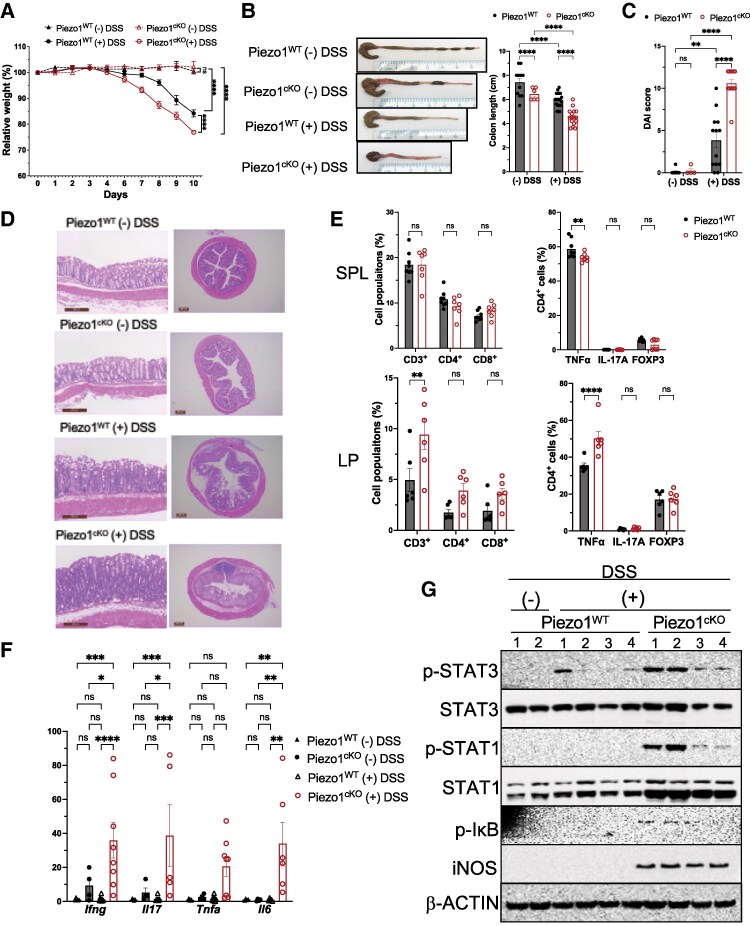
Deletion of Piezo1in T cells accelerates DSS-induced colitis. Chemically induced colitis occurred by introducing 2% DSS in drinking water of mice for 7 d, followed by normal tap water for 3 d. The control group received only normal tap water throughout the experiment. (A) Percent weight loss of Piezo1^cKO^ mice after DSS treatment. Percent weight defined as follows: (final weight – initial weight)/initial weight × 100, *n* = 8 (Piezo1^WT^ – DSS), 5 (Piezo1^cKO^ − DSS), 14 (Piezo1^WT^ + DSS), and 16 (Piezo1^cKO^ + DSS). (B) The DAI was measured; DAI = weight loss score + stool character score + hematochezia score; score 0 represents no disease symptoms, and score 12 represents the most severe symptoms. *n* = 8 (Piezo1^WT^ − DSS), 13 (Piezo1^cKO^ − DSS), 4 (Piezo1^WT^ + DSS), and 13 (Piezo1^cKO^ + DSS). (C) Representative photos of colons from mice and quantification of colon length (cm) at day 10. *n* = 6 (Piezo1^WT^ − DSS), 16 (Piezo1^cKO^ − DSS), 11 (Piezo1^WT^ + DSS), and 20 (Piezo1^cKO^ + DSS). (D) H&E staining of the colon 10 d after DSS treatment. Scale bar = 200 µm. (E) Flow cytometry analysis of T-cell subpopulations in LP and SPL 10 d after DSS treatment (*n* = 6 to 8). (F) Relative *Ifnγ*, *Tnfα*, *Il-17*, *Il-6*, and cytokine mRNA expression in LP of Piezo1^WT^ or Piezo1^cKO^ mice with or without DSS treatment (*n* = 4 to 9). (G) Expression of phospho-STAT3, STAT3, phospho-STAT1, STAT1, phospho-IκB, and inducible nitric oxide synthase in LP was determined by Western blot of colon mucosa from Piezo1^WT^ and Piezo1^cKO^ mice with or without DSS treatment. β-ACTIN was used as the loading control. Data were analyzed by 2-way analysis of variance with Tukey post hoc test (A, B, C, F) and with Šídák post hoc test (E). All bar graphs are shown as mean ± SEM with *P < 0.05, **P < 0.01, ***P < 0.001, and ****P < 0.0001.

CD4^+^ T cells are known as a key initiator of the intestinal inflammation process.^41^ Specifically, IL-17–producing CD4^+^ T cells are essential to the pathogenesis of IBD.^42^ Since our data have indicated that deletion of Piezo1 upregulates proinflammatory signatures in T cells, including IL-17 and related cytokines, we examined the function of Piezo1 on intestinal inflammation in vivo using an acute colitis mouse model. For this model, we chemically induced colitis by introducing 2% DSS in drinking water for 7 d and then exposed the mice to normal tap water for 3 d. Piezo1^cKO^ mice showed significantly more weight loss compared to Piezo1^WT^ mice after 10 d (Fig. 3A). DSS-treated Piezo1^cKO^ mice had a significantly higher DAI score and significantly increased colon shortening compared to DSS-treated Piezo1^WT^ mice (Fig. 3B and C). Cross-sectioning of colons from DSS-treated Piezo1^cKO^ mice showed marked inflammation, including immune cell infiltration, epithelial hyperplasia, and ulceration (Fig. 3D). Analysis of the LP showed a significant increase in the percentage of CD3^+^ T cells in DSS-treated Piezo1^cKO^ mice as compared with that of Piezo1^WT^ mice (Fig. 3E). In the CD4^+^ T-cell compartment, Piezo1^cKO^ mice had a significantly higher percentage of TNFα-expressing CD4^+^ T cells compared to Piezo1^WT^ CD4^+^ T cells in the LP and SPL, with no differences in either IL-17A^+^ cells or FOXP3-expressing cells in both SPL and LP (Fig. 3E). We also observed an increase in the relative mRNA expression of inflammatory cytokines *Ifnγ*, *Tnfα*, *Il-17*, and *Il-6* in the intestinal mucosa of Piezo1^cKO^ compared to Piezo1^WT^ (Fig. 3F) as well as the activation of the associated intracellular mediators of their response, including total STAT1, NF-κB, and STAT3 in the DSS-treated Piezo1^cKO^ (Fig. 3G). The phosphorylation of STAT1 and STAT3 was increased in the colon of DSS-treated Piezo1^cKO^ mice, whereas phospho-STAT1 was undetectable in DSS-treated Piezo1^WT^ mice (Fig. 3G). Iκb phosphorylation was also detected in the intestinal mucosa of Piezo1^cKO^ mice compared with those of DSS-treated Piezo1^WT^ mice. As expected, inducible nitric oxide synthase expression was also increased in the intestinal mucosa of Piezo1^cKO^ mice compared with those of DSS-treated Piezo1^WT^ mice (Fig. 3G), which is a common phenomenon of intestinal inflammation.^43^

### 3.4 Deletion of Piezo1 in T cells diminishes EAE severity

To further explore the role of Piezo1 in regulating T-cell function in vivo, we utilized the EAE mouse model. EAE is a T-cell–driven autoimmune disease, and it is the most commonly used experimental model of the human inflammatory demyelinating disease, multiple sclerosis.^44^ Active EAE was induced in both Piezo1^WT^ or Piezo1^cKO^ mice by immunization of myelin oligodendrocyte peptide fragment 35–55 (MOG_35–55_) emulsified in CFA on day 0 followed by injection of pertussis toxin 2 h and 24 h later. In both Piezo1^WT^ or Piezo1^cKO^, clinical scores progressively increased to a peak between days 16 and 18 and slowly decreased until plateauing at day 24 (Supplementary Fig. 3A and B). By day 16, Piezo1^cKO^ mice had a significantly decreased clinical score compared to their Piezo1^WT^ counterparts, which was sustained until the end of the experiment. On day 25, the spleens and inguinal lymph nodes (LNs) were harvested to assess differences in the CD4^+^ T-cell populations. Overall, the frequency of CD4^+^ T cells found in the spleen did not vary between Piezo1^WT^ and Piezo1^cKO^ mice; interestingly, however, there was an increase in CD4^+^ T cells found in the LNs of Piezo1^cKO^ mice (Supplementary Fig. 3C). Furthermore, there was no significant difference in the percentage of IFNγ- or IL-17A–expressing CD4^+^ T cells in the spleen or lymph nodes (Supplementary Fig. 3D). Surprisingly, we observed a statistically significant increase in the naive compartment (CD44^−^CD62L^+^) and a decrease in the effector memory compartment (CD44^+^CD62L^lo^) in the LNs of Piezo1^cKO^ mice (Supplementary Fig. 3E). No difference was seen in the central memory compartment of CD4^+^ T cells. This trend could be seen in the spleen as well, although it was not statistically significant (Supplementary Fig. 3E). Taken together, unlike the direct DSS-induced colitis model, our data suggest that Piezo1 deficiency in CD4^+^ T cells significantly decreases the clinical severity of the active EAE disease model with a reduction of CD4^+^ effector memory cells in the draining lymph nodes (DLNs) during chronic stimulation.

**Fig. 4.**
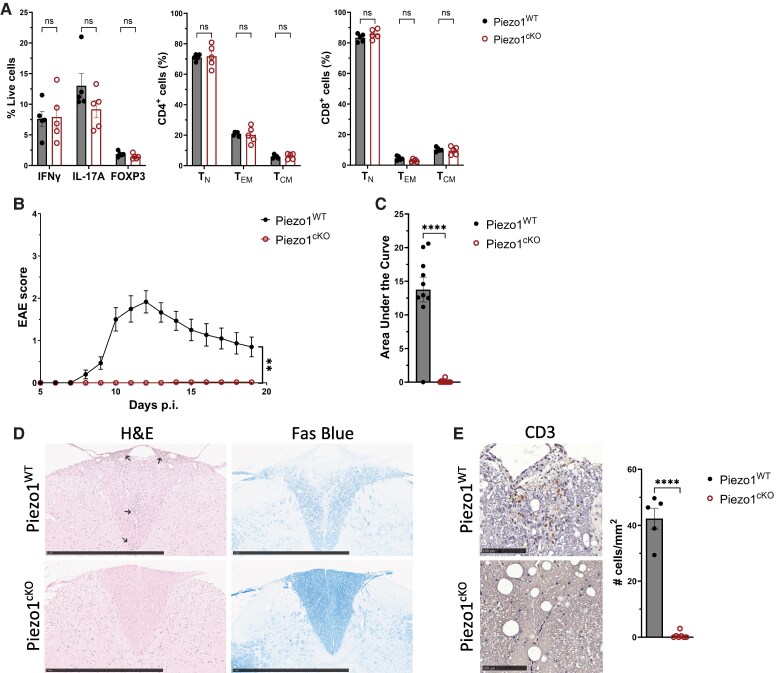
Deletion of Piezo1 in T cells diminishes EAE severity. CD45.1 mice were injected intraperitoneally with 20 million encephalitogenic in vitro restimulated splenocytes from MOG-immunized Piezo1^WT^ or Piezo1^cKO^ mice, as described in the AT-EAE protocol. (A) Flow cytometry analysis of the relative frequency of IFNγ-, IL-17A-, and FOXP3-expressing CD4^+^ T-cell and naive (T_N_: CD44^−^CD62L^+^), effector memory (T_EM_: CD44^+^CD62L^−^), and central memory (T_CM_: CD44^+^CD62L^+^) populations of the in vitro stimulated splenocytes with 20 μg MOG_35–55_ peptide and rmIL-23 (20 ng/mL) for 3 d before adoptive transfer (*n* = 5). (B) Mean clinical scores showing progression of adoptive transfer EAE in CD45.1 recipients injected with indicated donor cells (*n* = 10). (C) Severity of EAE calculated as area under the curve (AUC) of the clinical score (*n* = 10). (D) Representative H&E and Fast Blue staining of the spinal cords at 12 DPI. Scale bar = 1 mm (*n* = 3). (E) Representative immunohistochemistry staining and quantification of CD3^+^ cells found in the spinal cord at 20 DPI. Scale bar = 100 µm (*n* = 5).

**Fig. 5.**
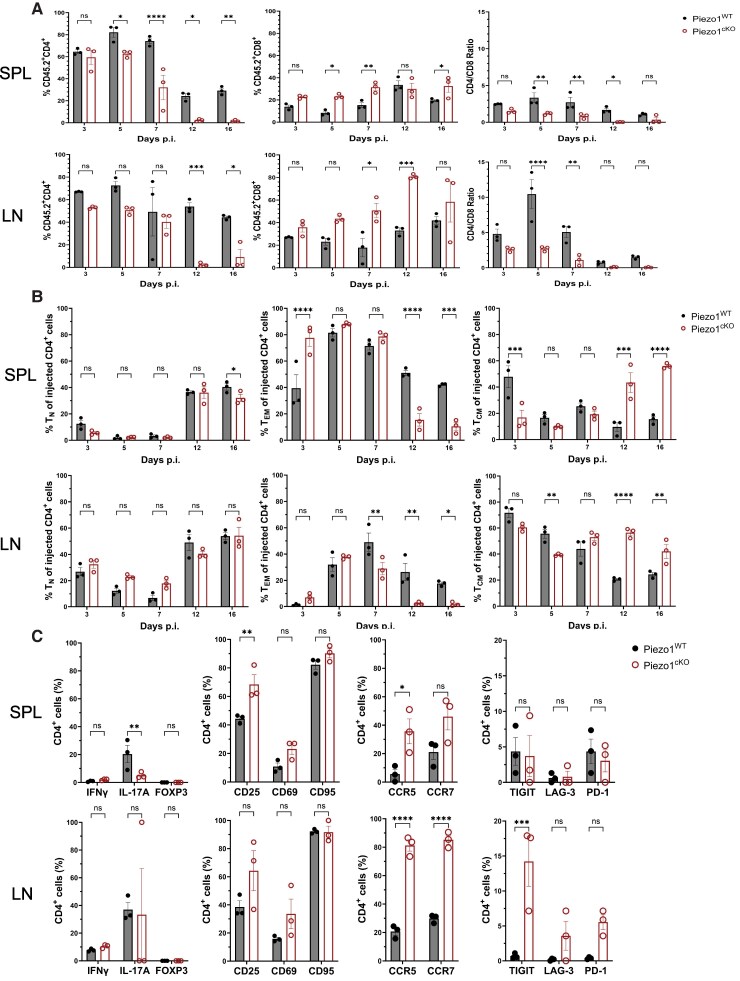
Deletion of Piezo1 in T cells diminishes TEM cells in EAE. CD45.1 mice were injected intraperitoneally with 20 million encephalitogenic in vitro restimulated splenocytes from MOG-immunized Piezo1^WT^ or Piezo1^cKO^ mice, as described in the AT-EAE protocol. (A) Flow cytometry analysis of the relative frequency and ratio of CD4- and CD8-expressing donor (CD45.2^+^) cells from the SPL and draining LN at 3, 5, 7, 12, and 16 DPI (*n* = 3). (B) Flow cytometry analysis of the relative frequency of CD4^+^ T-cell naive (T_N_: CD44^−^CD62L^+^), effector memory (T_EM_: CD44^+^CD62L^−^), and central memory (T_CM_: CD44^+^CD62L^+^) populations from the SPL and draining LN at 3, 5, 7, 12, and 16 DPI (*n* = 3). (C) Flow cytometry analysis of the relative frequency of CD4^+^ T cells expressing key cytokines (IFNγ, IL-17A, and FOXP3), activation markers (CD25, CD69, and CD95), chemokines (CCR5 and CCR7), and exhaustion markers (TIGIT, LAG-3, and PD-1) from the SPL and draining LN at 3, 5, 7, 12, and 16 DPI (*n* = 3). Data were analyzed by 2-way analysis of variance with Tukey post hoc test (a–g). All bar graphs are shown as mean ± SEM with *P < 0.05, **P < 0.01, ***P < 0.001, and ****P < 0.0001.

As the primary active EAE model does not allow a mechanistic delineation between effector T-cell homing, function, and persistence in the central nervous system (CNS) vs ongoing generation from the naive T-cell subset in the immunized LN in EAE pathogenesis, we utilized the AT-EAE to further investigate the role of Piezo1 on the encephalitogenic potential of effector CD4^+^ T-cell function. Donor Piezo1^WT^ or Piezo1^cKO^ mice were immunized with MOG_35–55_ peptide, and 11 d later, splenocytes from these immunized mice were harvested and incubated in vitro with MOG_35–55_ peptide and rmIL-23 to expand MOG_35–55_–specific T_H_17 effector T cells. These pathogenic T_H_17 cells were adoptively transferred into wild-type C57BL/6 (CD45.1) recipient mice, allowing for long-term tracking of the pathogenic donor cells. Although MOG_35–55_–expanded Piezo1^cKO^ CD4^+^ T cells before recipient transfer produced similar amounts of proinflammatory cytokines IFNγ and IL-17A and had similar frequencies of naive and memory phenotypes as the Piezo1^WT^ control (Fig. 4A), unexpectedly, the Piezo1^cKO^ MOG_35–55_–expanded cells were unable to elicit neuroinflammation in recipient CD45.1 mice following the adoptive transfer (Fig. 4B and C). At peak disease 12 d after adoptive T-cell injection (DPI), Piezo1^WT^ mice showed lymphocytic infiltration into the dorsal column of the spinal cord accompanied by a profound demyelination within the dorsal column of the spinal cords (Fig. 4D). In contrast, Piezo1^cKO^ mice showed neither lymphocytic infiltration nor demyelination at 12 DPI (Fig. 4D). At 20 DPI, immunohistochemistry showed sustained lymphocytic infiltration in Piezo1^WT^ spinal cords with 29 to 50 more CD3^+^ T cells per millimeter of tissue; in contrast, Piezo1^cKO^ recipient mice showed less than 5 CD3^+^ T cells per millimeter of tissue in the spinal cord (Fig. 4E). To investigate why Piezo1^cKO^ CD4^+^ effector cells were incapable of inducing disease, a time course was performed to examine the T-cell populations in the spleen and DLNs of the recipient mice before expected disease onset (days 3 and 5), at disease onset (day 7), at peak disease (day 12), and at steady-state disease (day 16). Interestingly, Piezo1 deficiency selectively decreased the CD4^+^ T-cell numbers, leading to a significantly reduced CD4^+^/CD8^+^ ratio (Fig. 5A). Analysis of the T-cell subpopulations revealed that the Piezo1 deficiency drastically decreased the effector memory CD4^+^ T cells and increased central memory CD4^+^ T cells in the spleen and DLN by 12 DPI (Fig. 5B). This impact on the effector memory population was not seen in the CD8^+^ population; in fact, there were more Piezo1^cKO^ CD8^+^ effector memory T cells than Piezo1^WT^ in the spleen throughout the course of disease (Supplementary Fig. 5A). Notably, we could not detect any effector memory CD4^+^ Piezo1^cKO^ T cells in DLN by 12 DPI, which could explain the significant decrease in IL-17A–expressing Piezo1^cKO^ CD4^+^ T cells compared to Piezo1^WT^ (Fig. 5B). Further investigation of the Piezo1-deficient CD4^+^ T-cell population showed an increase in expression of activation markers CD25 and CD69, chemokines CCR5 and CCR7, and exhaustion markers TIGIT, LAG-3, and PD-1 (Fig. 5C). The opposite effect was seen in the Piezo1-deficient CD8^+^ T-cell population, displaying a decrease in the same markers (Supplementary Fig. 5B). Taken together, these data support our prior finding that Piezo1 impacts the functional roles in CD4^+^ T cells that are not seen in CD8^+^ T cells.^33^ Our data further highlight the importance of Piezo1 in restraining CD4^+^ T-cell differentiation and persistence of the effector memory subpopulation.

Since AT-EAE requires immunization followed by in vitro restimulation, we examined how Piezo1-depleted CD4^+^ T cells respond to antigen rechallenge. We immunized transgenic OT-II Piezo1^cKO^ mice and OT-II Piezo1^WT^ mice with OVA_323–329_ in CFA emulsion intradermally at the base of the tail. At 10 DPI and 44 DPI, CD4^+^ T cells were stimulated in vitro with irradiated splenocytes pulsed with OVA_323–329_. At 10 DPI, Piezo1^cKO^ CD4^+^ T cells showed minimal differences in activation and exhaustion markers. In the draining LN, there was a decrease in effector memory T cells, with a subsequent increase in the central memory population similar to the results seen in AT-EAE (Supplementary Fig. 6A). Antigenic rechallenge at 44 DPI showed no notable differences in activation, exhaustion, or memory development for Piezo1^cKO^ T cells compared to Piezo1^WT^ (Supplementary Fig. 6B). These results confirm that Piezo1 does not lead to any intrinsic deficiency in CD4^+^ T-cell activation, including in the setting of antigen rechallenge.

### 3.5 Deletion of Piezo1 in T cells prevents GvHD

**Fig. 6.**
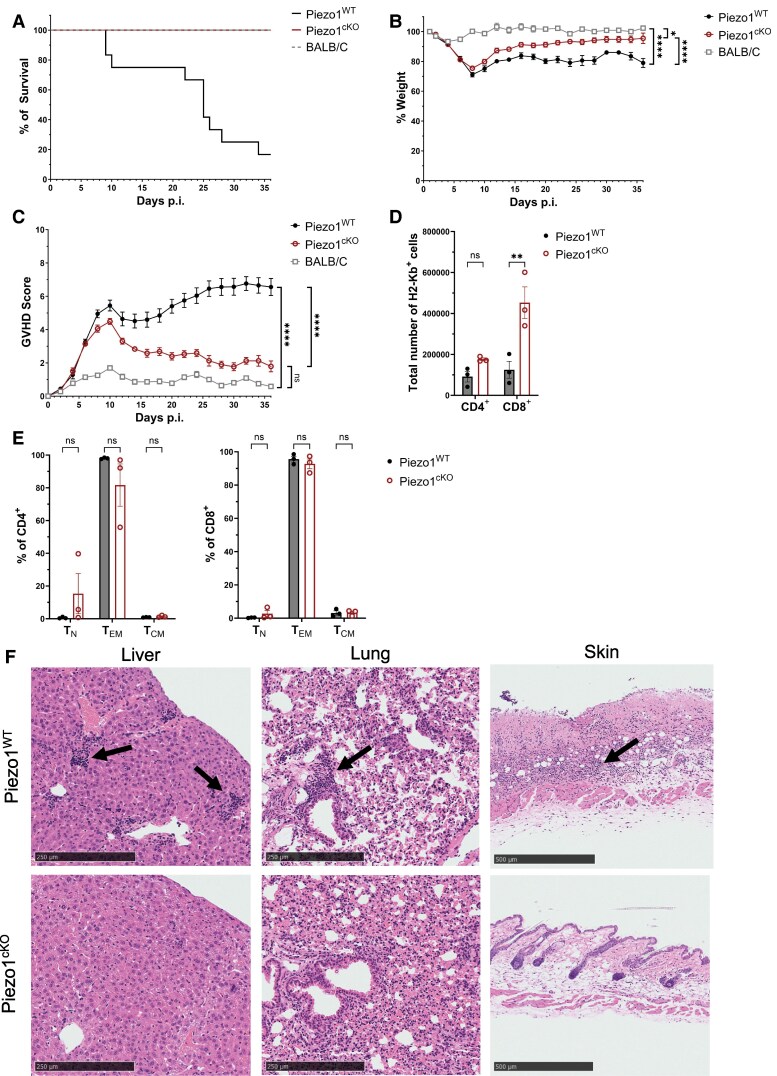
Deletion of Piezo1 in T cells diminishes GvHD severity. BALB/c host mice after transfer of allogenic T cells from Piezo1^WT^ or Piezo1^cKO^ donor mice. BALB/c mice were irradiated twice with 4 Gy and transplanted with 5 × 10^6^ allogenic C57BL/6 Piezo1^WT^ bone marrow (BM) or Piezo1^cKO^ BM cells together with 1 × 10^6^ allogenic CD4^+^ T cells from Piezo1^WT^ or Piezo1^cKO^ mice. (A) Survival, (B) weight loss, and (C) clinical scores of GvHD pathology (0 to 10) after transfer of allogenic T cells from Piezo1^WT^ or Piezo1^cKO^ donor mice. BALB/c T-cell and BM transfer were used as control (*n* = 7). (D) Total number of CD4^+^H2-Kb^+^ or CD8^+^H2-Kb^+^ cells in spleen 27 d after allogenic transfer of Piezo1^WT^ or Piezo1^cKO^ T cells. (E) Flow cytometry analysis of the relative frequency of CD4^+^ and CD8^+^ T-cell naive (T_N_: CD44^−^CD62L^+^), effector memory (T_EM_: CD44^+^CD62L^−^), and central memory (T_CM_: CD44^+^CD62L^+^) populations at 27 DPI. (F) Representative H&E-stained microsections of the liver, lung, and skin of BALB/c host mice 27 d after allogenic transfer of Piezo1^WT^ or Piezo1^cKO^ T cells. Scale bar = 200 µm. Data were analyzed by 2-way analysis of variance with Tukey post hoc test (A–F). All bar graphs are shown as mean ± SEM with *P < 0.05, **P < 0.01, ***P < 0.001, and ****P < 0.0001.

To investigate the role of Piezo1 in naive T cells, we utilized another model of CD4^+^ T-cell transfer-induced pathology. GvHD, induced by the reaction of donor T cells to recipient histo-incompatible antigens in the transplant recipient, is a serious complication of allogenic bone marrow transplantation.^45^ Irradiated BALB/c mice were transplanted with 5 × 10^6^ bone marrow cells and whole splenocytes containing 1 × 10^6^ CD4^+^ T cells from either Piezo1^WT^ or Piezo1^cKO^ mice in the B6 background. The resulting major histocompatibility complex (MHC) mismatch results in a robust CD4^+^ T-cell–mediated GvHD. Mice transplanted with Piezo1^WT^ cells developed severe GvHD as demonstrated by poor survival (Fig. 6A), more severe and persistent weight loss (Fig. 6B), and worse clinical scores (Fig. 6C) as compared to Peizo1^cKO^ mice. Interestingly, mice transplanted with Piezo1^cKO^ cells initially had similar weight loss and clinical scores as Piezo1^WT^-transplanted mice over the first 7 to 10 d after transplant (Fig. 6B and C), but they then recovered to the same levels as syngeneic transplanted controls. Additionally, mice transplanted with Piezo1^cKO^ cells did not experience the same level of lethality as seen in the Piezo1^WT^-transplanted mice (Fig. 6A). At 27 d posttransplant (DPT), there were more H2-Kb–transplanted CD4^+^ and CD8^+^ T cells seen in the spleens of mice transplanted with Piezo1^cKO^ than those transplanted with Piezo1^WT^ cells (Fig. 6D). Within the CD4^+^ T-cell population, Piezo1^cKO^ T cells had a lower percentage of effector memory cells compared to Piezo1^WT^, which was not seen in the CD8^+^ population, albeit it was not statistically significant (Fig. 6E). Although there were more Piezo1^cKO^ T cells in the spleens compared to Piezo1^WT^, clusters of infiltrating lymphocytes could only be seen in the liver, lung, and skin tissues of mice transplanted with Piezo1^WT^ cells but not Piezo1^cKO^ at 27 DPT (Fig. 6F).

### 3.6 Piezo1-deficient T cells fail to induce colitis after adoptive naive CD4^+^ T-cell transfer

**Fig. 7.**
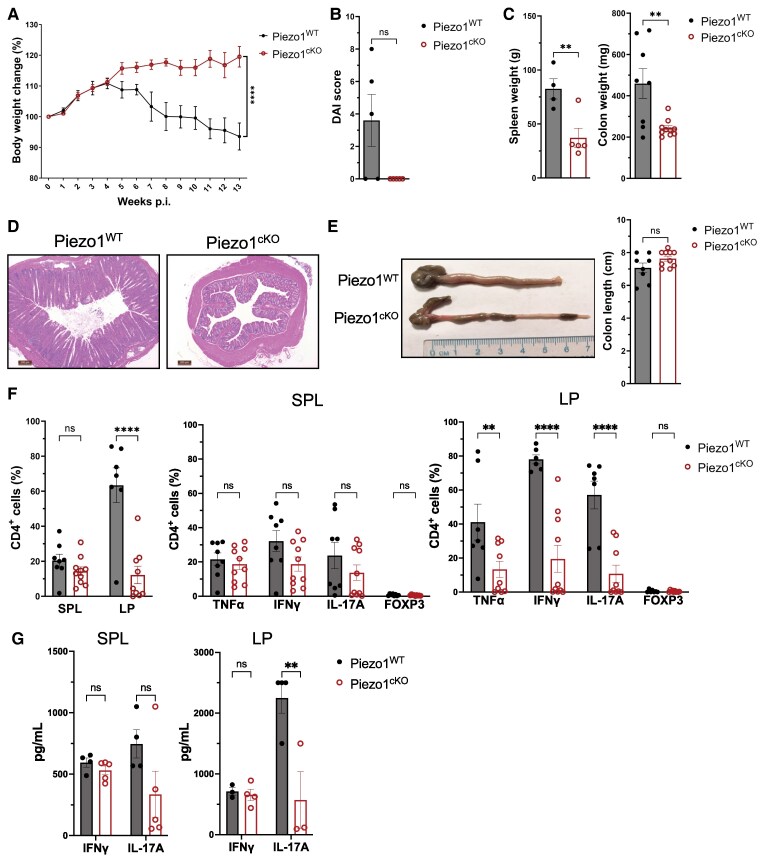
Deletion of Piezo1 in T cells fails to induce colitis after adoptive transfer. Rag1^−/−^ mice were transferred with naive CD4^+^CD45Rb^hi^ T cells from Piezo1^WT^ or Piezo1^cKO^ mice. (A) Weight loss curves as a percentage of original body weight. *n* = 6 (Piezo1^WT^), *n* = 10 (Piezo1^cKO^). (B) The DAI was measured; DAI =weight loss score + stool character score + hematochezia score; score 0 represents no disease symptoms, and score 12 represents the most severe symptoms (*n* = 10). (C) Spleen (*n* = 4 Piezo1^WT^, *n* = 5 Piezo1^cKO^) and colon (*n* = 8 Piezo1^WT^, *n* = 10 Piezo1^cKO^) weight (g) was measured at 13 WPI from mice described in A. (D) Representative H&E staining of the colon sections as shown in A. Scale bar = 200 µm. (E) Representative photos and quantification of the length of colon samples from mice described in A. (F) Flow cytometry analysis of CD4^+^ T cells isolated from spleen and LP of Rag1^−/−^ mice 13 WPI of Piezo1^WT^ or Piezo1^cKO^ naive T cells and stimulated ex vivo with PMA and ionomycin for 6 h (*n* = 6 to 8). (G) CD4^+^ T cells isolated from spleen and LP of Rag1^−/−^ mice 13 WPI of Piezo1^WT^ or Piezo1^cKO^ naive T cells and stimulated ex vivo with PMA and ionomycin for 48 h. IFNγ and IL-17A expression was determined by an enzyme-linked immunosorbent assay (*n* = 3 to 5). Data were analyzed by 2-way analysis of variance with Šídák post hoc test (A) and Tukey post hoc test (B–G). All bar graphs are shown as mean ± SEM with *P < 0.05, **P < 0.01, ***P < 0.001, and ****P < 0.0001.

Our results thus far have shown a stark contrast in disease phenotype between DSS-induced colitis, in which the affected pathologic tissue is in local proximity to sites of T-cell activation, and EAE and GvHD, in which affected tissues are distant from main sites of T-cell activation. To further assess the consistency of Piezo1 impact on effector T-cell differentiation and persistence in vivo, we revisited the colitis model and employed an adoptive transfer model of chronic intestinal inflammation that requires induction of transferred naive CD4^+^ T-cell migration, activation, and effector function for pathogenesis.^46,47^ Naive CD4^+^ T cells (CD4^+^CD45^RBhigh^) from Piezo1^WT^ or Piezo1^cKO^ were injected into Rag1^−/−^ mice to induce intestinal inflammation. Rag^−/−^ mice that received T cells from Piezo1^WT^ mice lost approximately 10% of their original body weight and had an increased DAI score, while Rag^−/−^ mice that received T cells from Piezo1^cKO^ mice showed no weight loss or disease phenotype (Fig. 7A and B). Consistent with disease severity, the colons of Piezo1^WT^ T-cell–transferred mice showed shortening, thickening, and bleeding. In contrast, the colons of mice that were injected with Piezo1^cKO^ T cells appeared completely healthy (Fig. 7C and E). Furthermore, the spleens of Piezo1^WT^ recipient mice at week 13 were significantly larger than those of Piezo1^cKO^ recipient mice, indicating an increase in chronic inflammation (Fig. 7C). H&E staining of the colons at week 13 showed increased immune cell infiltration, epithelial hyperplasia, and ulceration only in mice that received Piezo1^WT^ T cells (Fig. 7D). Consistent with reduced colitis, the frequency of CD4^+^ T cells in the LP were significantly reduced in mice that had received T cells from Piezo1^cKO^ mice compared to Piezo1^WT^, while no differences were observed in the spleen (Fig. 7F). There was also a significant decrease in TNFα-, IFNγ-, and IL-17A–producing CD4^+^ T cells in the LP of Piezo1^cKO^ compared with that of Piezo1^WT^ transferred mice, while no detectable difference was observed in FOXP3^+^ CD4^+^ T cells in the LP of recipient mice (Fig. 7F). Upon restimulation ex vivo, Piezo1^cKO^ donor T cells isolated from the LP secreted significantly less IL-17A compared to their Piezo1^WT^ counterparts (Fig. 7G). Similar results were also seen in Piezo1^cKO^ donor T cells isolated from the spleen but to a lesser extent. Interestingly, no difference was seen in the release of IFNγ upon ex vivo restimulation in the LP of Piezo1^WT^ and Piezo1^cKO^ T-cell transferred mice (Fig. 7G).

**Fig. 8.**
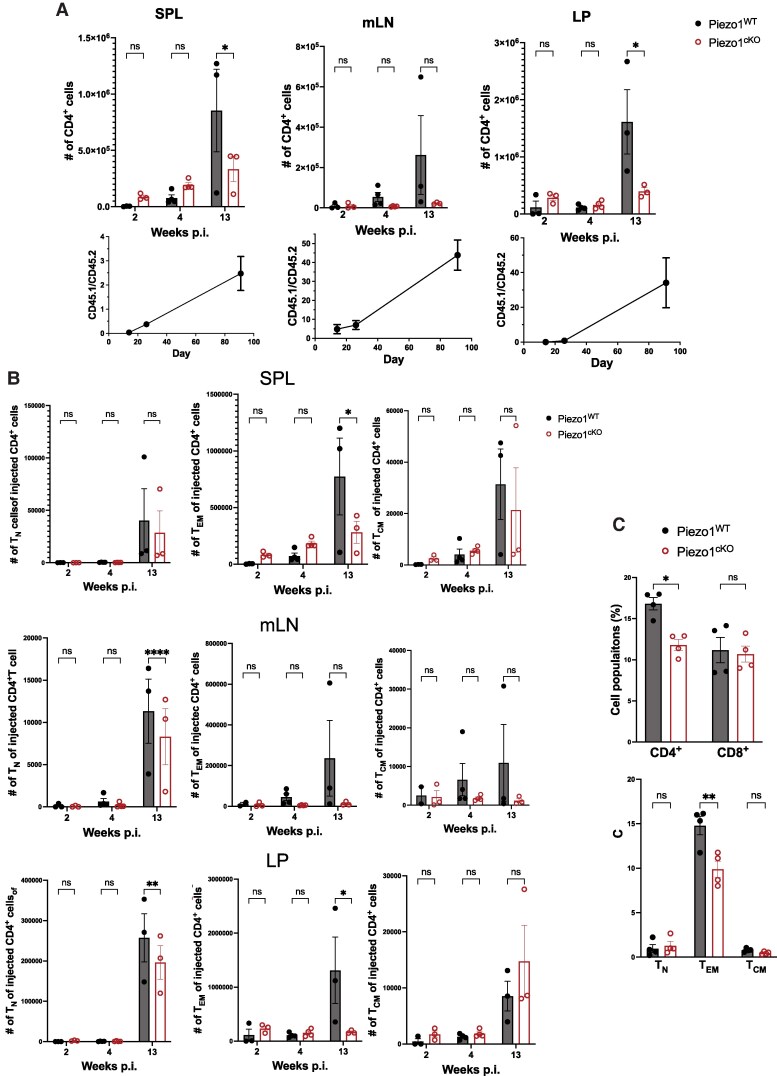
Deletion of Piezo1 in T cells diminishes T_EM_ cells in adoptive transfer-induced colitis. Rag1^−/−^ mice were adoptively cotransferred with naive CD4^+^ T cells isolated from Piezo1^WT^ mice (CD45.1) and Piezo1^cKO^ mice (CD45.2) at a ratio of 1:1 (5×10^5^:5×10^5^). Recipients were sacrificed 2, 4, or 13 wk after transfer (*n* = 3 to 4). (A) The number of C57BL/6 CD4^+^ T cells (CD45.1^+^) and Piezo1^cKO^ CD4^+^ T cells (CD45.2^+^) in the SPL, mLN, and colon (LP) at 2, 4, and 13 wk postinjection as analyzed by flow cytometry. (B) Flow cytometry analysis of the number of CD4^+^ T-cell naive (T_N_: CD44^−^CD62L^+^), effector memory (T_EM_: CD44^+^CD62L^−^), and central memory (T_CM_: CD44^+^CD62L^+^) populations from the SPL, mLN, and LP at 2, 4, and 13 wk postinjection. (C) Top: the frequency of CD4^+^ and CD8^+^ populations in the spleen of 2-yr-old Piezo1^WT^ or Piezo1^cKO^ mice. Bottom: the frequency of effector memory (T_EM_: CD4^+^CD44^+^CD62L^−^), central memory (T_CM_: CD4^+^CD44^+^CD62L^+^), and naive cells (T_N_: CD4^+^CD44^−^CD62L^+^) in spleens of 2-yr-old Piezo1^WT^ or Piezo1^cKO^ mice (*n* = 4). Data were analyzed by 2-way analysis of variance with Tukey post hoc test (A–C). All bar graphs are shown as mean ± SEM with *P < 0.05, **P < 0.01, ***P < 0.001, and ****P < 0.0001.

Altogether, our results have shown that Piezo1^cKO^ CD4^+^ T cells failed to induce naive CD4^+^ T-cell–induced pathology such as EAE, colitis, and GvHD in vivo. To exclude the complication of absent inflammation in the Piezo1^cKO^ transferred group, we cotransferred CD45.1 Piezo1^WT^ and CD45.2 Piezo1^cKO^ T cells at a 1:1 ratio into Rag1^−/−^ mice. Two, 4, or 13 wk postinjection (WPI), CD45.1 and CD45.2 T-cell populations in the spleen, mLN, and LP were measured. Only Piezo1^WT^ CD45.1 T-cell populations showed a time-dependent increase in the spleen, mLN, and LP. By 13 WPI, only Piezo1^WT^ CD45.1 T cells were predominantly found, and not much accumulation of CD45.2 Piezo1^cKO^ T cells was detected in SPL, mLN, and LP (Fig. 8A). Therefore, the ratio between CD45.1 (Piezo1^WT^) and CD45.2 (Piezo1^cKO^) in the SPL, mLN, and LP was time-dependently increased with a ratio of 43.8 and 34.1 at 13 WPI in mLN and LP, respectively (Fig. 8A). In addition, the number of naive T (T_N_) cells of Piezo1^cKO^ (CD45.2) was significantly lower compared to that of Piezo1^WT^ (CD45.1) in the mLN and LP by 13 WPI, and T_EM_ cells of Piezo1^cKO^ (CD45.2) in the same tissues were not increased by 13 WPI (Fig. 8B). Furthermore, longitudinal studies of 2-yr-old Piezo1^cKO^ mice showed a significantly lower frequency of splenic CD4^+^ T cells as compared with that of Piezo1^WT^; however, we did not observe any difference in the frequency of CD8 T cells between aged Piezo1^cKO^ and Piezo1^cKO^ mice. In particular, the number of the effector memory (CD44^+^CD62L^−^) CD4^+^ T-cell subset was significantly diminished compared to that of Piezo1^WT^ (Fig. 8C), suggesting effector memory T-cell generation is dependent upon Piezo1 expression. Altogether, our observations support the view that Piezo1 expression in CD4^+^ T cells is essential for the effector memory T-cell persistence, contributing to the pathogenesis of CD4^+^ T-cell–mediated immunopathology while restraining pathogenic T_H_1 and T_H_17 development.

## 4. Discussion

In the present study, we find that Piezo1 expression in CD4^+^ T cells does not impact the proliferation or expression of activation markers CD25 and CD69 upon anti-CD3/CD28 stimulation. Under polarizing conditions alongside anti-CD3/CD28 stimulation, Piezo1 restrains proinflammatory CD4^+^ differentiation into T_H_1 and T_H_17 cells but does not impact Treg differentiation, in sharp contrast to a previous report utilizing the same animal model.^37^ GSEA and qPCR analyses revealed that Piezo1^cKO^ T cells had increased mRNA levels of pathogenic T_H_17 cell signatures, IFNγ signaling, and inflammatory responses. During DSS-colitis, Piezo1^cKO^ mice had more severe disease and showed increased signs of inflammation within the LP, confirming the hyperinflammatory potential of Piezo1^cKO^ T cells in vivo compared to the Piezo1^WT^ controls. Surprisingly, in chronic autoimmune mouse models of EAE, Piezo1^cKO^ mice had less severe disease, and transfer of MOG-specific pathogenic T_H_17-differentiated Piezo1^cKO^ T cells were incapable of inducing disease. Cell tracking throughout the course of AT-EAE revealed that Piezo1^cKO^ CD4^+^ T cells were activated to the same degree or more than Piezo1^WT^ T cells. Interestingly, our data suggest that the lack of disease induction is due, in part, to the decreased persistence of the effector memory CD4^+^ population. In an IBD mouse model of naive T-cell transfer, Piezo1^cKO^ T cells did not induce inflammation in the colons of the recipients. Again, cell tracking again revealed that there is a decreased persistence of the CD4^+^ effector memory pool. Collectively, these observations suggest that Piezo1 is essential for effector T-cell persistence, and without a continuous pool of newly activated CD4^+^ T cells, these Piezo1^cKO^ T cells are incapable of inducing phenotypic autoimmune diseases.

Mechanosensation by T cells, via mechanosensitive ion channels, including Piezo1, occurs in a variety of physiologic and pathologic states, transmitting physical and mechanical information within a cellular and tissue environment into a biochemical signaling cascade affecting T-cell function and biology. One form of such mechanical forces is the shear stress experienced by circulating T cells in the vasculature. Recent studies have shed light on the important functional role of shear stress–induced Piezo1 function in enhancing T-cell receptor (TCR)-mediated T-cell activation and overall fitness,^48,49^ suggesting that employing long-term shear stress or pharmacologic activation of Piezo1 may result in enhanced and sustained T-cell activation following TCR stimulation, which may represent a potential translational opportunity to enhance the T cellular therapy manufacturing process for clinical application. Using T cells with genetic deletion of Piezo1 in the current study, we confirmed the specific contribution of Piezo1 in maintaining the long-term fitness of the effector memory T-cell pool following TRC stimulation. Additionally, we were also able to investigate the specific functional impact of Piezo1 in naive T cells in addition to TCR-activated T cells, in the absence of compensatory Piezo2 overexpression.^34^

While prior studies applied shear stress and TCR stimulation to result in enhanced T-cell activation using in vitro model systems,^48,49^ canonical TCR activation on naive T cells typically occurs in secondary lymphoid organs such as the LN and spleen, where shear stress is minimal, whereas reexposure of antigen-experienced T cells to cognate MHC/peptide complexes on APCs or target tissues happens either in the tissues—where shear stress is minimal—or on the apical surfaces of endothelial or damaged tissues exposed to intravascular contents under pathological conditions. We have previously examined the intravascular adhesive and migration behavior of leukocytes in postcapillary venules and observed that extensional stress—created due to changes in vessel geometry and directly implicated lateral membrane stretching—contributes critically to leukocyte behavior beyond that attributed by shear stress.^50^ Indeed, the in vivo contexts in which T cells use Piezo1 activation are multifactorial and complex and require additional in vivo and in vitro investigations using different model systems to address varying sources of mechanotransducing membrane deformation in vivo, such as physical contact forces, intercellular pressure, and extracellular matrix stiffness.

Another scenario where mechanosensation impacts T-cell functional states is in the formation of the immunological synapse with APC or the target cells.^36^ Even though T-cell recognition of cognate antigen is known to be subjected to mechanical forces, little is known about Piezo1's potential role in T-cell receptor function and downstream outcomes. Studies of Piezo1 in human T cells have shown its peripheral distribution around the immunological synapse.^36^ Further, knocking out Piezo1 via CRISPR resulted in decreased expression of pZAP70, CD69, and CD25 by flow.^36^ Interestingly, our studies in Piezo1^cKO^ mouse T cells have shown no impact on markers of activation, including CD69 and CD25 when Piezo1 is depleted. A similar study of Piezo1 in murine T cells showed parallel results that Piezo1^cKO^ mice have less severe autoimmune disease. However, the authors of these studies contributed these findings to an increase in Treg differentiation in Piezo1^cKO^ CD4^+^ T cells.^37^ Curiously, we have been unable to find differences in Treg polarization across all of our inflammatory mouse models and during in vitro polarization. These differences could be the result of varying microbiota impacting the inflammatory responses or slight variations in the experimental protocols. These discrepant results highlight the need for a mechanistic understanding of Piezo1's role during T-cell activation.

Intriguingly, our results suggest that Piezo1 deletion does not impact CD8^+^ T-cell function, in strong agreement with our prior studies utilizing the same T-cell lineage-specific Piezo1 deletion mouse model in the setting of solid tumors.^34^ Studies have shown that treatment with MOG_35–55_ pMHCII-CAR T-cell therapy during active EAE at the time of disease onset markedly limited disease activity, which corresponded with a decrease in MOG_38–49_ tetramer^+^ cells in the CNS.^51^ Similar to our results, this suggests that the effector CD4^+^ T cells are a necessary contributor to EAE pathogenesis, regardless of the presence of Treg cells.

In summary, our study underscores an important role of Piezo1 in CD4^+^ T-cell function during acute and chronic inflammatory mouse models. Although Piezo1^cKO^ T cells show increased proinflammatory markers during in vitro stimulation, they are unable to induce chronic disease. Given the striking impact on adoptive transfer models of inflammation and a primary decrease in effector memory populations of Piezo1^cKO^ CD4^+^ T cells, our data support the view that Piezo1 contributes to the persistence of effector CD4^+^ T cells.

## Supplementary Material

qiae242_Supplementary_Data

## Data Availability

The original contributions presented in the study are included in the article/Supplementary material. Further inquiries can be directed to the corresponding authors.

## References

[1] Chen Y, Ju L, Rushdi M, Ge C, Zhu C. Receptor-mediated cell mechanosensing. Mol Biol Cell. 2017:28(23):3134–3155. 10.1091/mbc.E17-04-0228 28954860 PMC5687017

[2] Ingber DE . Cellular mechanotransduction: putting all the pieces together again. FASEB J. 2006:20(7):811–827. 10.1096/fj.05-5424rev 16675838

[3] Zhu C, Chen W, Lou J, Rittase W, Li K. Mechanosensing through immunoreceptors. Nat Immunol. 2019:20(10):1269–1278. 10.1038/s41590-019-0491-1 31534240 PMC7592628

[4] Du H, Bartleson JM, Butenko S, Alonso V, Liu WF, Winer DA, Butte MJ. Tuning immunity through tissue mechanotransduction. Nat Rev Immunol. 2023:23(3):174–188. 10.1038/s41577-022-00761-w 35974148 PMC9379893

[5] Rushdi M, Li K, Yuan Z, Travaglino S, Grakoui A, Zhu C. Mechanotransduction in T cell development, differentiation and function. Cells. 2020:9(2):364. 10.3390/cells9020364 32033255 PMC7072571

[6] Wahl A, Dinet C, Dillard P, Nassereddine A, Puech PH, Limozin L, Sengupta K. Biphasic mechanosensitivity of T cell receptor-mediated spreading of lymphocytes. Proc Natl Acad Sci U S A. 2019:116(13):5908–5913. 10.1073/pnas.1811516116 30850545 PMC6442626

[7] Cinamon G, Shinder V, Alon R. Shear forces promote lymphocyte migration across vascular endothelium bearing apical chemokines. Nat Immunol. 2001:2(6):515–522. 10.1038/88710 11376338

[8] Saitakis M, Dogniaux S, Goudot C, Bufi N, Asnacios S, Maurin M, Randriamampita C, Asnacios A, Hivroz C. Different TCR-induced T lymphocyte responses are potentiated by stiffness with variable sensitivity. Elife. 2017:6:e23190. 10.7554/eLife.23190 28594327 PMC5464771

[9] Liu Y, Zhang T, Zhang H, Li J, Zhou N, Fiskesund R, Chen J, Lv J, Ma J, Zhang H, et al Cell softness prevents cytolytic T-cell killing of tumor-repopulating cells. Cancer Res. 2021:81(2):476–488. 10.1158/0008-5472.CAN-20-2569 33168645

[10] Chen W, Zhu C. Mechanical regulation of t-cell functions. Immunol Rev. 2013:256(1):160–176. 10.1111/imr.12122 24117820 PMC3818107

[11] Harrison DL, Fang Y, Huang J. T-cell mechanobiology: force sensation, potentiation, and translation. Front Phys. 2019:7:45. 10.3389/fphy.2019.00045 32601597 PMC7323161

[12] Dolmetsch RE, Lewis RS, Goodnow CC, Healy JI. Differential activation of transcription factors induced by ca2+ response amplitude and duration. Nature. 1997:386(6627):855–858. 10.1038/386855a0 9126747

[13] Hogan PG, Lewis RS, Rao A. Molecular basis of calcium signaling in lymphocytes: stim and orai. Annu Rev Immunol. 2010:28(1):491–533. 10.1146/annurev.immunol.021908.132550 20307213 PMC2861828

[14] Vig M, Kinet J-P. Calcium signaling in immune cells. Nat Immunol. 2009:10(1):21–27. 10.1038/ni.f.220 19088738 PMC2877033

[15] Premack BA, Gardner P. Signal transduction by t-cell receptors: mobilization of ca and regulation of ca-dependent effector molecules. Am J Physiol. 1992:263(6):C1119–C1140. 10.1152/ajpcell.1992.263.6.C1119 1282295

[16] Feske S . Calcium signalling in lymphocyte activation and disease. Nat Rev Immunol. 2007:7(9):690–702. 10.1038/nri2152 17703229

[17] McCarl C-A, Khalil S, Ma J, Oh-hora M, Yamashita M, Roether J, Kawasaki T, Jairaman A, Sasaki Y, Prakriya M, et al Store-operated ca2+ entry through orai1 is critical for T cell-mediated autoimmunity and allograft rejection. J Immunol. 2010:185(10):5845–5858. 10.4049/jimmunol.1001796 20956344 PMC2974040

[18] Kaufmann U, Kahlfuss S, Yang J, Ivanova E, Koralov SB, Feske S. Calcium signaling controls pathogenic th17 cell-mediated inflammation by regulating mitochondrial function. Cell Metab. 2019:29(5):1104–1118.e6. 10.1016/j.cmet.2019.01.019 30773462 PMC6506368

[19] Oh-hora M, Rao A. Calcium signaling in lymphocytes. Curr Opin Immunol. 2008:20(3):250–258. 10.1016/j.coi.2008.04.004 18515054 PMC2574011

[20] Dombroski JA, Hope JM, Sarna NS, King MR. Channeling the force: piezo1 mechanotransduction in cancer metastasis. Cells. 2021:10(11):2815. 10.3390/cells10112815 34831037 PMC8616475

[21] Murthy SE, Dubin AE, Patapoutian A. Piezos thrive under pressure: mechanically activated ion channels in health and disease. Nat Rev Mol Cell Biol. 2017:18(12):771–783. 10.1038/nrm.2017.92 28974772

[22] Song S, Zhang H, Wang X, Chen W, Cao W, Zhang Z, Shi C. The role of mechanosensitive piezo1 channel in diseases. Prog Biophys Mol Biol. 2022:172:39–49. 10.1016/j.pbiomolbio.2022.04.006 35436566

[23] Kamajaya A, Kaiser JT, Lee J, Reid M, Rees DC. The structure of a conserved piezo channel domain reveals a topologically distinct β sandwich fold. Structure. 2014:22(10):1520–1527. 10.1016/j.str.2014.08.009 25242456 PMC4192063

[24] Ge J, Li W, Zhao Q, Li N, Chen M, Zhi P, Li R, Gao N, Xiao B, Yang M. Architecture of the mammalian mechanosensitive piezo1 channel. Nature. 2015:527(7576):64–69. 10.1038/nature15247 26390154

[25] Coste B, Murthy SE, Mathur J, Schmidt M, Mechioukhi Y, Delmas P, Patapoutian A. Piezo1 ion channel pore properties are dictated by c-terminal region. Nat Commun. 2015:6(1):7223. 10.1038/ncomms8223 26008989 PMC4445471

[26] Coste B, Mathur J, Schmidt M, Earley TJ, Ranade S, Petrus MJ, Dubin AE, Patapoutian A. Piezo1 and piezo2 are essential components of distinct mechanically activated cation channels. Science. 2010:330(6000):55–60. 10.1126/science.1193270 20813920 PMC3062430

[27] Coste B, Xiao B, Santos JS, Syeda R, Grandl J, Spencer KS, Kim SE, Schmidt M, Mathur J, Dubin AE, et al Piezo proteins are pore-forming subunits of mechanically activated channels. Nature. 2012:483(7388):176–181. 10.1038/nature10812 22343900 PMC3297710

[28] Guo YR, MacKinnon R. Structure-based membrane dome mechanism for piezo mechanosensitivity. Elife. 2017:6:e33660. 10.7554/eLife.33660 29231809 PMC5788504

[29] Haselwandter CA, MacKinnon R. Piezo’s membrane footprint and its contribution to mechanosensitivity. Elife. 2018:7:e41968. 10.7554/eLife.41968 30480546 PMC6317911

[30] Haselwandter CA, Guo YR, Fu Z, MacKinnon R. Quantitative prediction and measurement of piezo’s membrane footprint. Proc Natl Acad Sci U S A. 2022:119(40):e2208027119. 10.1073/pnas.2208027119 36166475 PMC9546538

[31] Yang X, Lin C, Chen X, Li S, Li X, Xiao B. Structure deformation and curvature sensing of piezo1 in lipid membranes. Nature. 2022:604(7905):377–383. 10.1038/s41586-022-04574-8 35388220

[32] Mulhall EM, Gharpure A, Lee RM, Dubin AE, Aaron JS, Marshall KL, Spencer KR, Reiche MA, Henderson SC, Chew TL, et al Direct observation of the conformational states of piezo1. Nature. 2023:620(7976):1117–1125. 10.1038/s41586-023-06427-4 37587339 PMC10468401

[33] The Human Protein Atlas. https://www.proteinatlas.org/ENSG00000103335-PIEZO1/single+cell#immune_cell

[34] Abiff M, Alshebremi M, Bonner M, Myers JT, Kim BG, Tomchuck SL, Santin A, Kingsley D, Choi SH, Huang AY. Piezo1 facilitates optimal T cell activation during tumor challenge. Oncoimmunology. 2023:12(1):2281179. 10.1080/2162402X.2023.2281179 38126029 PMC10732680

[35] Pang R, Sun W, Yang Y, Wen D, Lin F, Wang D, Li K, Zhang N, Liang J, Xiong C, et al Piezo1 mechanically regulates the antitumour cytotoxicity of T lymphocytes. Nat Biomed Eng. 2024:8(9):1162–1176. 10.1038/s41551-024-01188-5 38514773

[36] Liu CSC, Raychaudhuri D, Paul B, Chakrabarty Y, Ghosh AR, Rahaman O, Talukdar A, Ganguly D. Cutting edge: piezo1 mechanosensors optimize human T cell activation. J Immunol. 2018:200(4):1255–1260. 10.4049/jimmunol.1701118 29330322

[37] Jairaman A, Othy S, Dynes JL, Yeromin AV, Zavala A, Greenberg ML, Nourse JL, Holt JR, Cahalan SM, Marangoni F, et al Piezo1 channels restrain regulatory T cells but are dispensable for effector CD4+ T cell responses. Sci Adv. 2021:7(28):eabg5859. 10.1126/sciadv.abg5859 34233878 PMC8262815

[38] Lei C, Sun R, Xu G, Tan Y, Feng W, McClain CJ, Deng Z. Enteric vip-producing neurons maintain gut microbiota homeostasis through regulating epithelium fucosylation. Cell Host Microbe. 2022:30(10):1417–1434.e8. 10.1016/j.chom.2022.09.001 36150396 PMC9588764

[39] Syeda R, Xu J, Dubin AE, Coste B, Mathur J, Huynh T, Matzen J, Lao J, Tully DC, Engels IH, et al Chemical activation of the mechanotransduction channel piezo1. Elife. 2015:4:e07369. 10.7554/eLife.07369 26001275 PMC4456433

[40] Wu X, Tian J, Wang S. Insight into non-pathogenic th17 cells in autoimmune diseases. Front Immunol. 2018:9:1112. 10.3389/fimmu.2018.01112 29892286 PMC5985293

[41] Bouma G, Strober W. The immunological and genetic basis of inflammatory bowel disease. Nat Rev Immunol. 2003:3(7):521–533. 10.1038/nri1132 12876555

[42] Fouser LA, Wright JF, Dunussi-Joannopoulos K, Collins M. Th17 cytokines and their emerging roles in inflammation and autoimmunity. Immunol Rev. 2008:226(1):87–102. 10.1111/j.1600-065X.2008.00712.x 19161418

[43] Kolios G, Valatas V, Ward SG. Nitric oxide in inflammatory bowel disease: a universal messenger in an unsolved puzzle. Immunology. 2004:113(4):427–437. 10.1111/j.1365-2567.2004.01984.x 15554920 PMC1782592

[44] Constantinescu CS, Farooqi N, O’Brien K, Gran B. Experimental autoimmune encephalomyelitis (eae) as a model for multiple sclerosis (ms). Br J Pharmacol. 2011:164(4):1079–1106. 10.1111/j.1476-5381.2011.01302.x 21371012 PMC3229753

[45] Shlomchik WD . Graft-versus-host disease. Nat Rev Immunol. 2007:7(5):340–352. 10.1038/nri2000 17438575

[46] Powrie F . T cells in inflammatory bowel disease: Protective and pathogenic roles. Immunity. 1995:3(2):171–174. 10.1016/1074-7613(95)90086-1 7648390

[47] Powrie F, Leach MW, Mauze S, Menon S, Barcomb Caddle L, Coffman RL. Inhibition of Th1 responses prevents inflammatory bowel disease in scid mice reconstituted with CD_45_RBhi CD4^+^ T cells. Immunity. 1994:1(7):553–562. 10.1016/1074-7613(94)90045-0 7600284

[48] Sarna NS, Desai SH, Kaufman BG, Curry NM, Hanna AM, King MR. Enhanced and sustained T cell activation in response to fluid shear stress. iScience. 2024:27(6):109999. 10.1016/j.isci.2024.109999 38883838 PMC11177201

[49] Hope JM, Dombroski JA, Pereles RS, Lopez-Cavestany M, Greenlee JD, Schwager SC, Reinhart-King CA, King MR. Fluid shear stress enhances T cell activation through piezo1. BMC Biol. 2022:20(1):61. 10.1186/s12915-022-01266-7 35260156 PMC8904069

[50] Benson BL, Li L, Myers JT, Dorand RD, Gurkan UA, Huang AY, Ransohoff RM. Biomimetic post-capillary venule expansions for leukocyte adhesion studies. Sci Rep. 2018:8(1):9328. 10.1038/s41598-018-27566-z 29921896 PMC6008471

[51] Yi J, Miller AT, Archambault AS, Jones AJ, Bradstreet TR, Bandla S, Hsu Y-S, Edelson BT, Zhou YW, Fremont DH, et al Antigen-specific depletion of CD4+ T cells by car T cells reveals distinct roles of higher- and lower-affinity tcrs during autoimmunity. Sci Immunol. 2022:7(76):eabo0777. 10.1126/sciimmunol.abo0777 36206355 PMC9867937

