# Supplementary material for: Piezo1 restrains proinflammatory response but is essential in T-cell–mediated immunopathology
Source: J Leukoc Biol. 2024 Nov 4;117(3):qiae242. doi: 10.1093/jleuko/qiae242 (PMC11953072; doi:10.1093/jleuko/qiae242)
Supplement: qiae242_Supplementary_Data [file qiae242_supplementary_data.docx]

Supplementary Material

Piezo1 restrains proinflammatory response but is essential in T cell-mediated immunopathology

**Sung Hee Choi^1,2*^, Alicia Santin^3*^, Jay T. Myers^2^, Byung-Gyu Kim^1,2^, Saada Eid^2^, Suzanne L. Tomchuck^2^, Daniel T. Kingsley^3^, Alex Y. Huang^1,2,3,4^**^†^

^1^Case Comprehensive Cancer Center, Case Western Reserve University School of Medicine, Cleveland, OH, U.S.A.

^2^Department of Pediatrics, Case Western Reserve University School of Medicine, Cleveland, OH, U.S.A.

^3^Department of Pathology, Case Western Reserve University School of Medicine, Cleveland, OH, U.S.A.

^4^Center for Pediatric Immunotherapy, Angie Fowler Adolescent and Young Adult Cancer Institute, University Hospitals Rainbow Babies & Children’s Hospital, Cleveland, OH, U.S.A.

^*^These authors contributed equally to this work and share first authorship.

^†^**Correspondence:**Alex Y. Huang, MD, PhD

WRB 6528, 2103 Cornell Road, Cleveland OH 44106-7288 U.S.A.

Email: Ayh3@case.edu; Phone: +1-216-368-1271

**Running title:** Piezo1 impact on CD4^+^ T cell function

**Total Supplementary Figures:** 7

**Supplementary Figure1.** Representative flow cytometry (left) and quantification (right) of proliferation of CD4^+^ T cells from Piezo1^WT^ and Piezo1^cKO^ mice. Purified CD4^+^ T cells were labeled with CFSE and activated with anti-CD3/ anti-CD28 beads for 3 days (*n*=6).

*
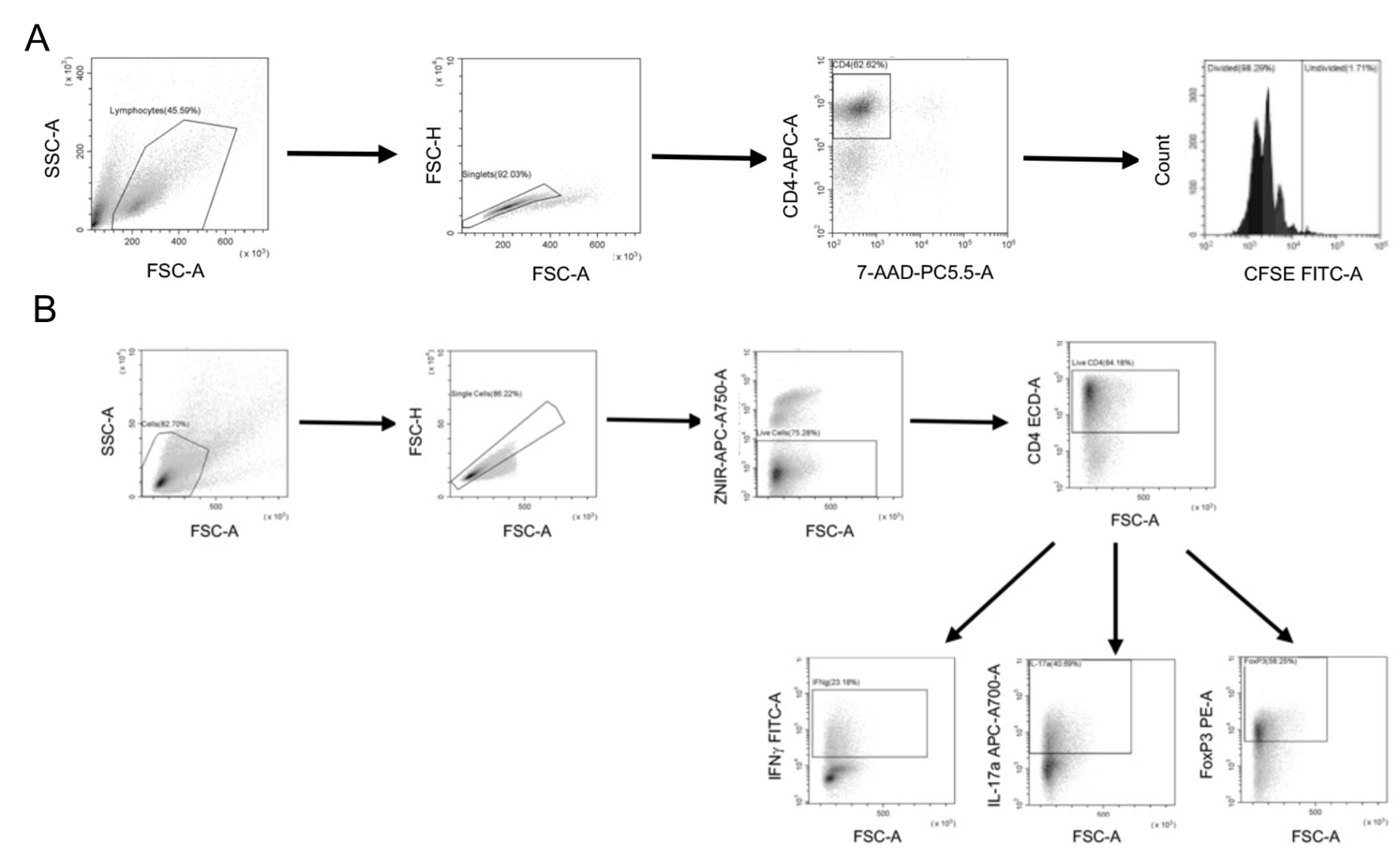
*

**Supplementary Figure 2**. Gating strategy to identify (A) CD4^+^ T cells cell proliferation (B) CD4^+^ T cells polarization from Piezo1^WT^ and Piezo1^cKO^ mice by conventional flow cytometric analysis.

**Supplementary Figure 3.** A) Mean clinical scores showing progression of active EAE in Piezo1^WT^ and Piezo1^cKO^ mice (*n*=10). B) Severity of EAE calculated as area under the curve (AUC) of the clinical score (*n*=10). C) Flow cytometry analysis of the relative frequency of CD4^+^ and CD8^+^ T cells, D) CD4^+^ T cell cytokine expression and E) CD4^+^ T naïve (T_N_: CD44^-^CD62L^+^), effector memory (T_EM_: CD44^+^CD62L^-^), and central memory (T_CM_: CD44^+^CD62L^+^) populations from the spleens (SPL) and draining lymph nodes (LN) at day 25 (*n*=5). Data were analyzed by two-way analysis of variance (ANOVA) with Tukey post hoc test (a-c). All bar graphs are shown as mean ± SEM with **P < 0.05, **P < 0.01, ***P < 0.001 and ****P < 0.0001.*

**
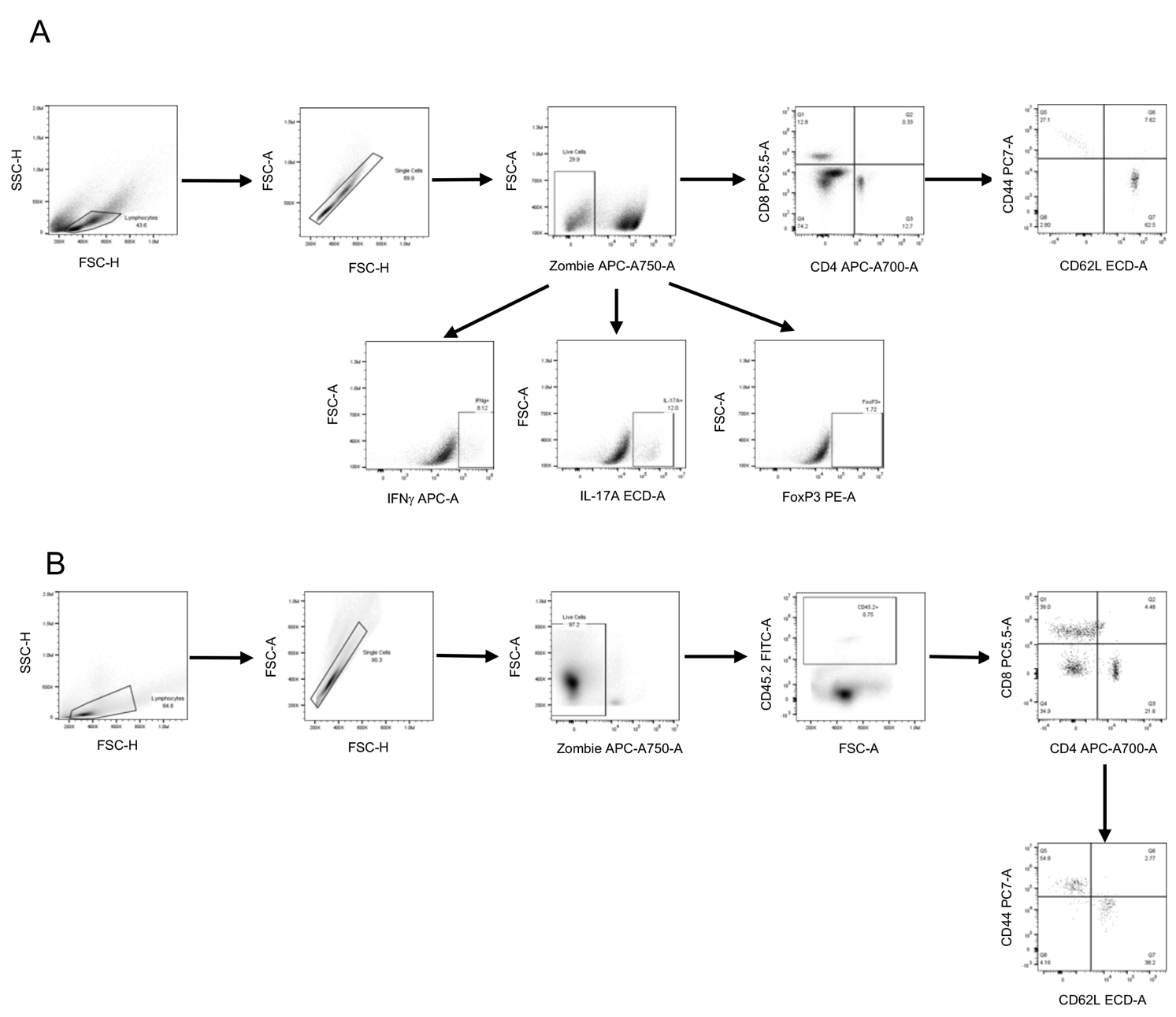
**

**Supplementary Figure 4.** Gating strategy to identify (A) T cell subpopulations in D3 encephalitogenic T cell cultures from Piezo1^WT^ or Piezo1^cKO^ mice. (B) CD45.2^+^ T cell subpopulations in spleens and lymph nodes at 3, 5, 7, 12, and 16 DPI of cultured Piezo1^WT^ or Piezo1^cKO^ encephalitogenic T cells into CD45.1^+^ mice.

**Supplementary Figure 5.** CD45.1 mice were injected intraperitoneal with 20 million encephalitogenic *in vitro* re-stimulated splenocytes from MOG-immunized Piezo1^WT^ or Piezo1^cKO^ mice as described in the AT-EAE protocol. A) Flow cytometry analysis of the relative frequency of CD8^+^ T cell naïve (T_N_: CD44^-^CD62L^+^), effector memory (T_EM_: CD44^+^CD62L^-^), and central memory (T_CM_: CD44^+^CD62L^+^) populations from the spleens (SPL) and draining lymph nodes (LN) at 3, 5, 7, 12, and 16 DPI (*n*=3). B) Flow cytometry analysis of the relative frequency of CD8^+^ T cells expressing key cytokines (IFNγ, IL-17A and FOXP3), activation markers (CD25, CD69, and CD95), chemokines (CCR5 and CCR7) and exhaustion markers (TIGIT, LAG-3, and PD-1) from the spleens (SPL) and draining lymph nodes (LN) at 12 DPI (*n*=3). Data were analyzed by two-way analysis of variance (ANOVA) with Tukey post hoc test (a-b). All bar graphs are shown as mean ± SEM with **P < 0.05, **P < 0.01, ***P < 0.001 and ****P < 0.0001.*

**

**Supplementary Figure 6.** OT-II Piezo1^WT^ and Piezo1^cKO^ mice were immunized with OVA_323-339_ in CFA intradermally at the base of the tail. A) Flow cytometry analysis of the relative frequency of activation markers (CD25, CD69, and CD95), T cell naïve (T_N_: CD44^-^CD62L^+^), effector memory (T_EM_: CD44^+^CD62L^-^), and central memory (T_CM_: CD44^+^CD62L^+^) populations, and exhaustion markers (TIGIT, LAG-3, and PD-1) before and after stimulation from the spleens (SPL) and draining lymph nodes (LN) at 10 DPI (*n*=4). B) Flow cytometry analysis of the relative frequency of activation markers (CD25, CD69, and CD95), T cell naïve (T_N_: CD44^-^CD62L^+^), effector memory (T_EM_: CD44^+^CD62L^-^), and central memory (T_CM_: CD44^+^CD62L^+^) populations, and exhaustion markers (TIGIT, LAG-3, and PD-1) before and after stimulation from the spleens (SPL) and draining lymph nodes (LN) at 44 DPI (Piezo1^WT^ *n*=3, Piezo1^cKO^ *n*=4). Data were analyzed by two-way analysis of variance (ANOVA) with Šídák post hoc test (a-b). All bar graphs are shown as mean ± SEM with **P < 0.05, **P < 0.01, ***P < 0.001 and ****P < 0.0001.*

**
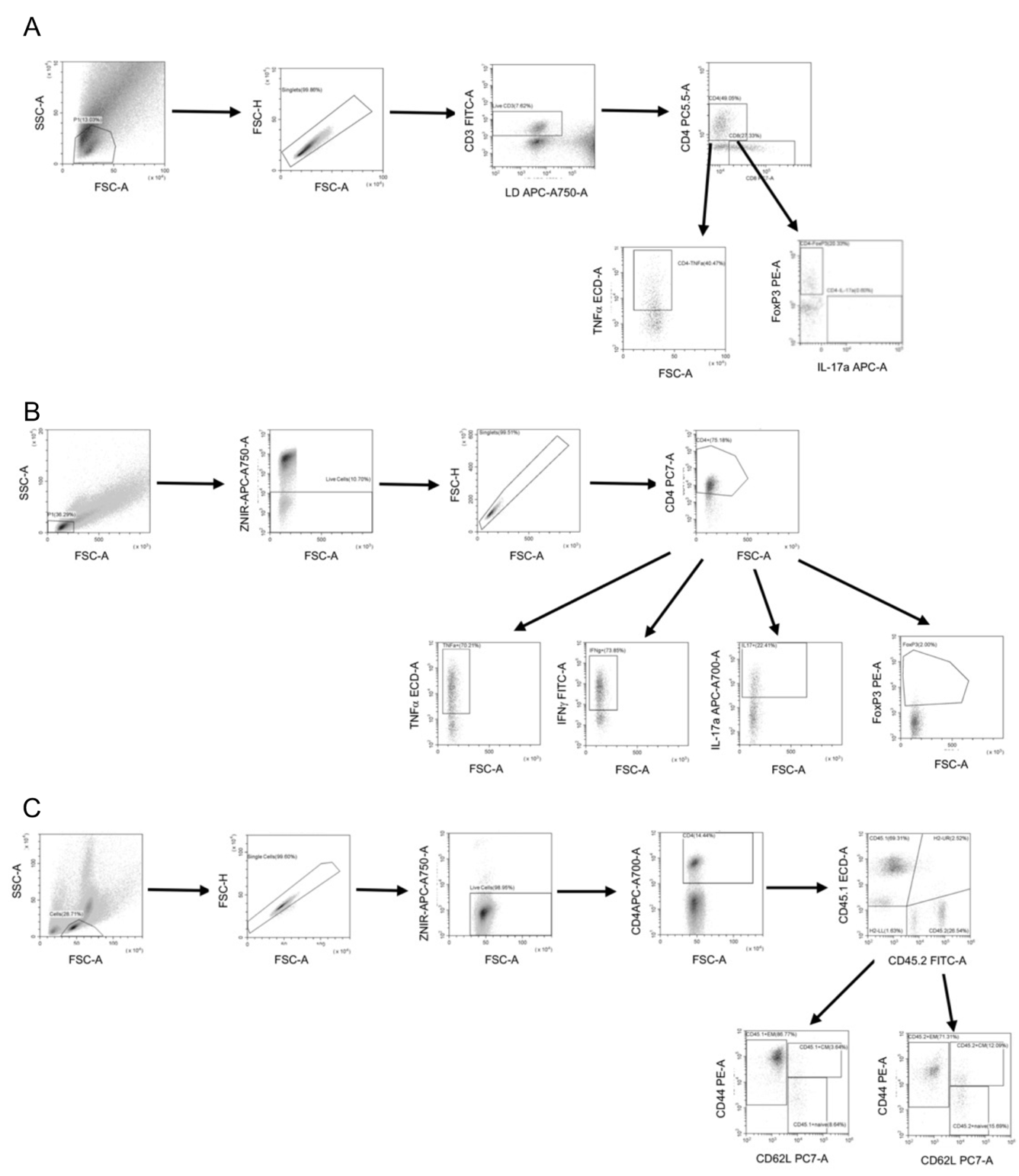
**

**Supplementary Figure 7**. Gating strategy to identify (A) T cell subpopulations in Lamina propria (LP) and spleen 10 days after DSS treatment (B) CD4^+^ T cells isolated from spleen and LP of Rag1^-/-^ mice 13 WPI of Piezo1^WT^ or Piezo1^cKO^ naive T cells (C) CD4^+^ T cell naïve (T_N_: CD44^-^CD62L^+^), effector memory (T_EM_: CD44^+^CD62L^-^), and central memory (T_CM_: CD44^+^CD62L^+^) populations from the spleen, mLN, and LP at 2, 4, and 13 weeks post injection by conventional flow cytometric analysis
